# A novel HSP90 inhibitor with reduced hepatotoxicity synergizes with radiotherapy to induce apoptosis, abrogate clonogenic survival, and improve tumor control in models of colorectal cancer

**DOI:** 10.18632/oncotarget.9774

**Published:** 2016-06-01

**Authors:** Linda Kinzel, Anne Ernst, Michael Orth, Valerie Albrecht, Roman Hennel, Nikko Brix, Benjamin Frey, Udo S. Gaipl, Gabriele Zuchtriegel, Christoph A. Reichel, Andreas Blutke, Daniela Schilling, Gabriele Multhoff, Minglun Li, Maximilian Niyazi, Anna A. Friedl, Nicolas Winssinger, Claus Belka, Kirsten Lauber

**Affiliations:** ^1^ Department of Radiation Oncology, Ludwig-Maximilians-University Munich, Munich, Germany; ^2^ Department of Radiation Oncology, Universitätsklinikum Erlangen, Friedrich-Alexander-Universität Erlangen-Nürnberg, Erlangen, Germany; ^3^ Department of Otorhinolaryngology, Head and Neck Surgery, and Walter Brendel Centre of Experimental Medicine, Ludwig-Maximilians-University Munich, Munich, Germany; ^4^ Institute of Veterinary Pathology at the Center for Clinical Veterinary Medicine, Ludwig-Maximilians-University Munich, Munich, Germany; ^5^ Department of Radiation Oncology, Klinikum rechts der Isar, Technische Universität München, Munich, Germany; ^6^ Department of Organic Chemistry, NCCR Chemical Biology, University of Geneva, Geneva, Switzerland

**Keywords:** HSP90 inhibition, radiosensitization, colorectal cancer, DNA damage response, cell death

## Abstract

The chaperone heat shock protein 90 (HSP90) crucially supports the maturation, folding, and stability of a variety of client proteins which are of pivotal importance for the survival and proliferation of cancer cells. Consequently, targeting of HSP90 has emerged as an attractive strategy of anti-cancer therapy, and it appears to be particularly effective in the context of molecular sensitization towards radiotherapy as has been proven in preclinical models of different cancer entities. However, so far the clinical translation has largely been hampered by suboptimal pharmacological properties and serious hepatotoxicity of first- and second-generation HSP90 inhibitors. Here, we report on NW457, a novel radicicol-derived member of the pochoxime family with reduced hepatotoxicity, how it inhibits the DNA damage response and how it synergizes with ionizing irradiation to induce apoptosis, abrogate clonogenic survival, and improve tumor control in models of colorectal cancer *in vitro* and *in vivo*.

## INTRODUCTION

Heat shock protein 90 (HSP90) is an ATP-dependent chaperone, which supports the folding, maturation, activation, and stability of a plethora of client proteins, including growth factor receptors, protein kinases, transcription factors, and others [1]. In cancer cells, this chaperoning function is of vital importance, since due to their aneuploidy cancer cells commonly suffer from proteotoxic stress, and mutant and/or overexpressed oncoproteins require intense folding assistance. If HSP90 function is perturbed, its client proteins are destined to proteasomal degradation. Accordingly, HSP90 inhibition has been identified as a multi-target approach of anti-cancer therapy, whose particular attractiveness derives from its capacity to affect multiple oncogenic pathways in parallel [2, 3].

The first HSP90 inhibitors described were the natural products geldanamycin (GA), a benzoquinone ansamycin antibiotic, and radicicol, a macrocyclic anti-fungal drug [4, 5]. However, due to severe toxicity, poor water solubility, and limited bioavailability, their preclinical evaluation was rapidly discontinued. Subsequently, they served as lead compounds for second-generation substances, such as 17-(Allylamino)-17-demethoxygeldanamycin (17-AAG), 17-Dimethyl-amino-ethyl-amino-17-demethoxy-geldanamycin (17-DMAG), NVP-AUY922, STA-9090 (Ganetespib), and others. The anti-cancer efficacy of these compounds has been extensively studied alone or in combination with classical chemotherapy, and/or targeted approaches, including protein kinase inhibitors and others [6–9]. On the basis of these encouraging preclinical results, 17 HSP90 inhibitors are currently undergoing clinical evaluation [10].

The variety of HSP90 client proteins reveal different sensitivities towards HSP90 inhibition, and proteomic analyses have disclosed that particular classes of proteins are preferentially affected [11]. Among the most susceptible proteins, regulators of the DNA damage response and protein kinases were identified. In accordance, radiotherapy appears as a specifically attractive partner for HSP90 inhibition in combined modality approaches [12]. Indeed, efficient radiosensitization of cancer cells has been reported in diverse cell culture systems *in vitro* [13–15]. However, the anti-tumor efficacy of HSP90 inhibition in combination with radiotherapy has rarely been examined *in vivo* and remains largely limited to xenograft models in immunocompromised mice [16–19].

In the present study, we utilized the novel HSP90 inhibitor NW457, a radicicol derivative of the pochoxime family, which was developed with specific focus on improved water solubility, bioavailability, and tolerability [20–23]. We focused on its potential applicability together with ionizing irradiation in models of colorectal carcinoma. Whereas radiotherapy constitutes a major treatment option for rectal cancer, its application for cancers of the colon remains limited to high-risk cases [24–26]. This is due to the rather high degree of mobility within this part of the large intestine and the adverse effects on the normal tissue if correspondingly large volumes with appropriate safety margins were irradiated. Hence, it is desirable to find substances which can sensitize the tumor tissue to irradiation and thus augment the therapeutic index. Employing different model systems of colorectal cancer, including human HCT116 cells, genetically modified subclones derived thereof, HCT8 cells, and mouse CT26 cells, we characterized the impact of NW457 on HSP90 client protein degradation, the DNA damage response, induction of different forms of cell death, senescence, and autophagy, as well as clonogenic survival *in vitro*, and evaluated its radiosensitizing potential and its tolerability *in vivo*. Although our mechanistic *in vitro* studies require further in depth analyses *in vivo*, our results so far identify NW457 as a potent radiosensitizer with convincing *in vivo* efficacy for combined modality approaches with ionizing irradiation.

## RESULTS

### NW457 is a potent HSP90 inhibitor with no detectable hepatocytotoxicity *in vitro*

As a proof of concept for HSP90 inhibition by the novel radicicol derivative NW457, we first analyzed the degradation of known HSP90 client proteins [27–29]. Human HCT116 colorectal cancer cells were treated with 0-300 nM NW457 for 24 h or 48 h, respectively, and the expression levels of ephrin A2 (EPHA2) and epidermal growth factor receptor (EGFR) were measured by FACS surface staining. Additionally, V-Raf murine sarcoma viral oncogene homologue B1 (BRAF) protein levels were assessed by Westernblot analyses of whole cell lysates. Incubation of human HCT116 colorectal cancer cells with NW457 clearly induced a dose- and time-dependent decrease in EPHA2, EGFR, and BRAF protein levels (Suppl. Figure 1A-1C), thus confirming its function as a potent HSP90 inhibitor. Apart from client protein degradation, upregulation of the heat shock response has been described as a central feature of HSP90 inhibition [15, 30]. Therefore, we next examined, whether this upregulation could also be observed in case of NW457. Quantitative realtime RT-PCR (qRT-PCR) analyses revealed a profound induction of HSP90 and HSP70 mRNA 12 h after NW457 treatment which was followed by an increase in HSP90 and HSP70 protein levels after 48 h (Suppl. Figure 1C-1D). In case of HSP70, this upregulation also translated into cell surface exposure and release into culture supernatants as determined by FACS surface staining and ELISA measurements (Suppl. Figure 1E-1F). Since the perspective of this study was to investigate the radiosensitizing potential of NW457, and radiosensitivity is known to depend on the cell cycle phase, we also performed cell cycle analyses and observed that treatment with 100 nM NW457 for 24 h leads to a reduction in S- and G2-phase cells paralleled by a slight accumulation of M- and G1-phase cells. Kinetically, this was preceded by a transient increase in G2- and M-phase cells after 6-12 h suggesting a prolongation of these cell cycle phases by NW457 treatment rather than a clear-cut cell cycle arrest at the known checkpoints (Suppl. Figure 1G-1H).

**Figure 1:**
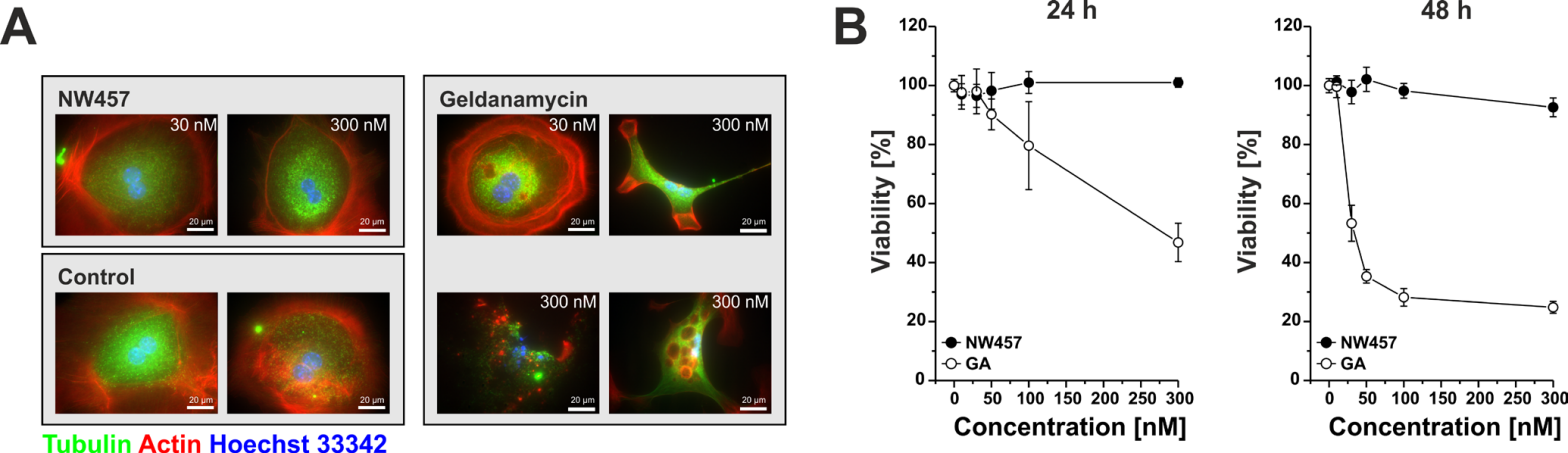
NW457 reveals no detectable hepatocytotoxicity *in vitro* Primary hepatocytes of C57BL/6 mice were exposed to 0-300 nM of NW457, geldanamycin (GA), or DMSO as vehicle control. Cellular morphology and viability were assessed by immunofluorescence microscopy and Alamar Blue assays. **A.** Immunofluorescence microscopy was performed after 24 h. Cells were fixed, permeabilized, and stained with anti-tubulin-FITC, phalloidin-Alexa Fluor 568 for actin visualization, and Hoechst 33342 for DNA visualization. Scale bars represent 20 μm. **B.** Alamar Blue viability tests were performed after 24 and 48 h, and the results were calibrated on the vehicle controls (100% viability). Means ± s.d. of intra-assay quadruplicates of one representative of three experiments are depicted.

We next analyzed the tolerability of NW457 by primary murine hepatocytes in comparison to GA, a first-generation HSP90 inhibitor whose hepatotoxic effects are well-known [31]. Cellular viability was examined by Alamar Blue assays 24-48 h after treatment, and hepatocellular morphology was monitored by immunofluorescence staining. Treatment with GA dramatically reduced the viability of primary hepatocytes in a time- and dose-dependent manner accompanied by severe morphological changes in the hepatocellular architecture (Figure 1). Features of apoptosis, including nuclear fragmentation and formation of apoptotic bodies were observed. Additionally, autophagic phenotypes with multiple intracellular vesicles appeared. In strong contrast, primary hepatocytes exposed to NW457 did not exhibit any of the mentioned characteristics, and even high inhibitor concentrations did not perturb the typical hepatocellular morphology. Concomitantly, the cellular viability remained virtually unaffected (Figure 1). Thus, NW457 appears to be a potent HSP90 inhibitor with no detectable toxicity on primary hepatocytes *in vitro*.

### NW457 synergizes with ionizing irradiation to induce chromatin condensation, nuclear fragmentation, apoptosis, and post-apoptotic, secondary necrosis

A number of HSP90 client proteins have been identified as key players in the cellular response to ionizing irradiation in tumor cells [17, 32]. Hence, pharmacological inhibitors of HSP90 are assumed to interfere with the radiation response *via* depletion of proteins associated with the relevant pathways. Notably, proteomic analyses revealed regulators of the DNA damage response to be most susceptible to HSP90 inhibition as compared to proteins of other signaling networks [11]. Therefore, we sought to characterize the radiosensitizing and cell death inducing effects of NW457 in combination with radiotherapy. According to our client protein degradation results (Suppl. Figure 1), we chose preincubation with NW457 for 24 h for all following combination experiments with ionizing irradiation. HCT116 cells were pretreated with NW457, irradiated at 5 Gy, and microscopical examination of nuclei was performed 24-72 h after irradiation. Typical apoptosis-associated morphological changes, including chromatin condensation and nuclear fragmentation, were observed upon treatment with NW457 alone in a time-dependent manner (Suppl. Figure 2A). Similar results were obtained for irradiation with 5 Gy alone. Notably, the combination of NW457 treatment and irradiation clearly potentiated these nuclear changes and strongly inhibited cell proliferation. Quantification of the microscopic data revealed a time-dependent, significant enhancement of chromatin condensation and nuclear fragmentation upon combined NW457 treatment plus irradiation *versus* irradiation or NW457 administration alone (Suppl. Figure 2B). In order to compare the potency of NW457 with a first-generation HSP90 inhibitor, GA was employed. NW457 showed similar potency of radiosensitization as GA (Suppl. Figure 2C).

**Figure 2:**
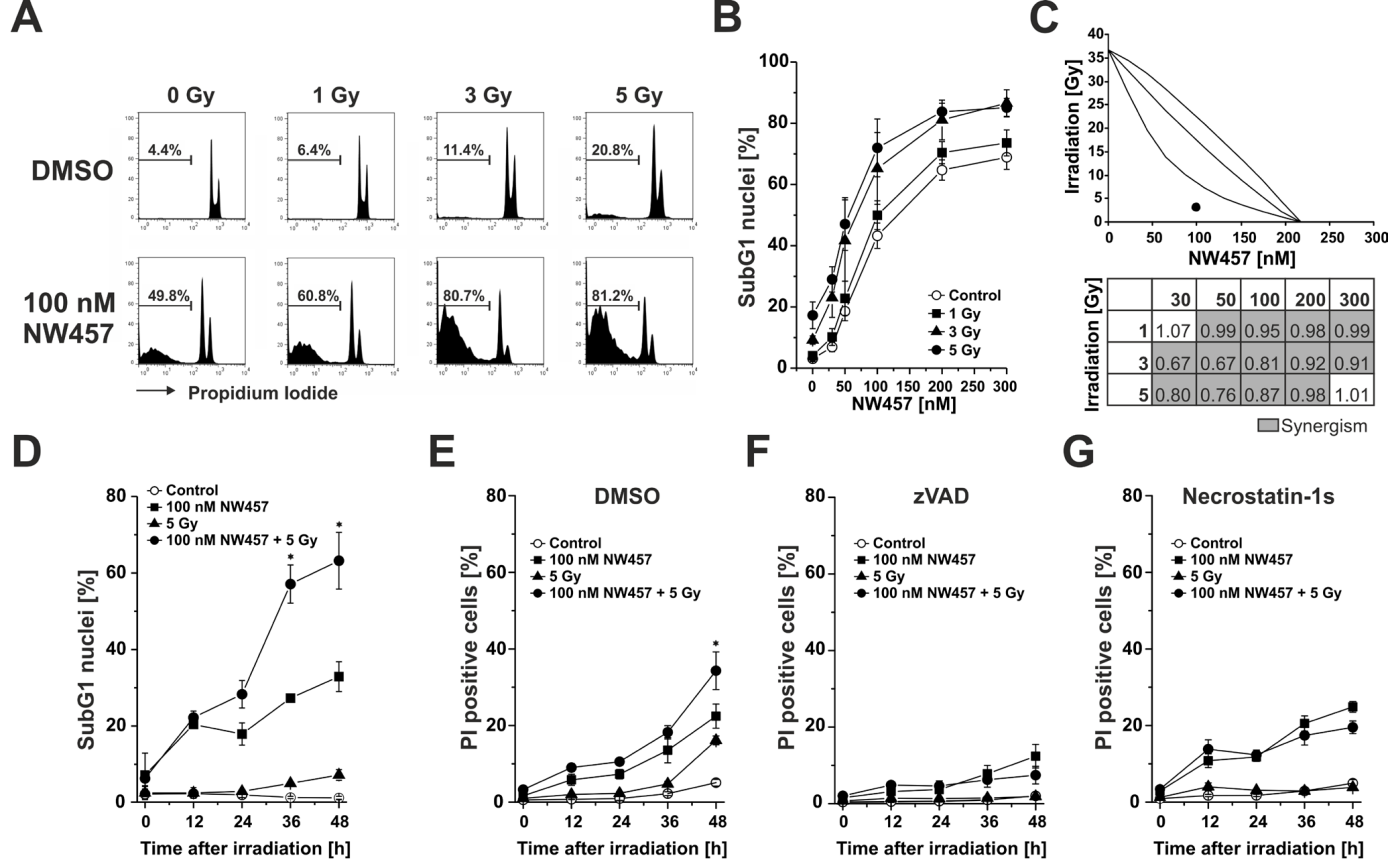
NW457 synergizes with ionizing irradiation to induce chromatin condensation, nuclear fragmentation, apoptosis, and post-apoptotic, secondary necrosis HCT116 cells were treated with 0-300 nM NW457 or DMSO as vehicle control for 24 h and irradiated at 0-5 Gy. Apoptosis induction was measured by FACS analysis of hypodiploid (subG1) nuclei 0-48 h after irradiation. **A.** Representative FACS histograms of the nuclear DNA content 48 h after irradiation. The percentage of subG1 nuclei is indicated. **B.** Dose-dependent formation of apoptotic subG1 nuclei after treatment with 0-300 nM NW457 and 0-5 Gy measured 48 h after irradiation. Means ± s.d. of four independent experiments are shown. **C.** NW457 synergizes with irradiation. *Upper panel:* Isobologram of the combination 100 nM NW457 and 3 Gy. The data point of the combined treatment lies below the surface of additivity showing a synergistic mode of action. *Lower panel:* Matrix of combination indices (CI) calculated from the data shown in (B). Values highlighted in grey (CI < 1) demonstrate synergism between NW457 and irradiation. **D.** Time-dependent formation of hypodiploid nuclei measured 0-48 h after irradiation at 5 Gy +/− treatment with 100 nM NW457. Means ± s.d. of three independent experiments are displayed. *p < 0.01 for combined treatment vs. irradiation only (unpaired Student's *t*-test). **E.**-**G.** Time course of plasma membrane disintegration assessed by PI exclusion FACS staining 0-48 h after irradiation at 5 Gy +/− treatment with 100 nM NW457 in the presence or absence of the poly-caspase inhibitor zVAD-fmk or the RIP kinase-1 inhibitor necrostatin-1s. *p < 0.05 for combined treatment vs. irradiation only (unpaired Student's *t*-test).

In parallel to our microscopic evaluation, the extent of NW457-induced DNA fragmentation was examined by flow cytometry. HCT116 cells were treated with 0-300 nM NW457 for 24 h, irradiated at 0-5 Gy, and 48 h after irradiation the nuclear DNA content was assessed by hypotonic propidium iodide (PI) staining and FACS analyses (Figure 2A). Whereas irradiation alone stimulated the appearance of hypodiploid nuclei only marginally, exposure to NW457 resulted in a strong and concentration-dependent increase, attaining a maximum at NW457 concentrations > 100 nM (Figure 2B). However, this effect was further elevated when the cells were additionally irradiated - a finding which again emphasizes the radiosensitizing potency of NW457.

In order to assess the quality of interaction (synergism, additivity, antagonism) between NW457 treatment and ionizing irradiation, isobologram analyses were performed, and combination indices (CI) were calculated for all combination treatments applied (Figure 2C) [33, 34]. 80% of the analyzed combinations showed synergistic effects (CI < 1, highlighted in grey), and these were predominantly observed for combinations comprising irradiation doses of 3 or 5 Gy, respectively. Comparable results were obtained with the isobologram method, which exhibits even higher stringency. A representative isobologram for the combination of 100 nM NW457 and 3 Gy is shown in Figure 2C. As the combined treatment with 50 or 100 nM NW457 plus 3-5 Gy irradiation resulted in the strongest synergistic effects, 100 nM was selected for further investigation of the kinetics of NW457-mediated radiosensitization. Exposure to 100 nM NW457 time-dependently increased the percentage of hypodiploid nuclei and significantly enhanced irradiation-induced DNA fragmentation when combined with 5 Gy (Figure 2D). At later stages, the cells underwent a delayed type of necrosis which was nearly quantitatively blocked by the poly-caspase inhibitor zVAD-fmk, thus identifying it as post-apoptotic, secondary necrosis [35–37] (Figure 2E-2F). A relevant contribution of necroptosis in this scenario could be excluded by using the receptor-interacting protein (RIP) kinase-1 inhibitor necrostatin-1s [35, 38] (Figure 2G). Taken together, these data strengthen our observations from the microscopic analyses and confirm the radiosensitizing capacity of NW457 in colorectal cancer cells.

### Enhanced apoptosis induction by NW457 in combination with irradiation is accompanied by increased caspase activation and caspase substrate cleavage

**Figure 3:**
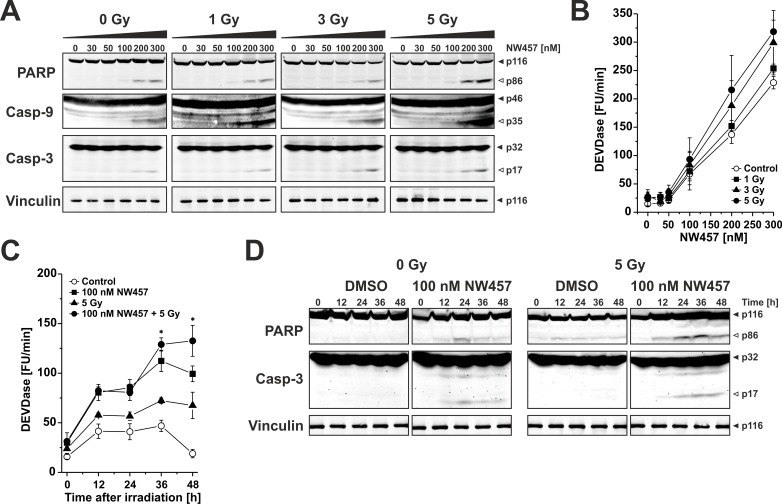
Enhanced apoptosis induction by NW457 in combination with irradiation is accompanied by increased caspase activation and caspase substrate cleavage Upon pretreatment with 0-300 nM NW457 for 24 h, HCT116 cells were irradiated at 0-5 Gy. Caspase activation and caspase substrate cleavage were monitored by Westernblot analyses and fluorometric caspase activity tests 0-48 h after irradiation. **A.** Dose response relationship of pro-caspase and caspase substrate processing upon treatment with 0-300 nM NW457 and 0-5 Gy measured 24 h after irradiation. Total protein extracts were separated *via* 6-15% SDS-PAGE and subjected to immunoblotting with antibodies against caspases-3, and −9, the caspase substrate PARP (150 μg protein per lane), or the loading control vinculin (10 μg protein per lane), respectively. Filled arrowheads indicate the full-length forms, and open arrowheads show the cleavage products. Representative blots of three independent experiments are depicted. **B.** Dose response relationship of caspase activation. Caspase activity was measured by fluorogenic DEVDase assays in protein lysates obtained from cells treated as in (A). Means ± s.d. of three independent experiments are shown. **C.** Time course analysis of DEVDase activity 0-48 h after irradiation with 5 Gy +/− pretreatment with 100 nM NW457 for 24 h. Data show means ± s.d. of one representative experiment performed in quadruplicates. *p < 0.05 for combined treatment vs. irradiation only (unpaired Student's *t*-test). **D.** Time dependence of proteolytic pro-caspase-3 processing and PARP cleavage (150 μg protein per lane) 0-48 h after irradiation. Vinculin served as loading control (10 μg protein per lane). Filled arrowheads indicate the full length forms, and open arrowheads depict the cleavage products. Representative blots of three independent experiments are shown.

Ionizing irradiation predominantly stimulates the intrinsic apoptotic pathway which is characterized by release of mitochondrial cytochrome c and subsequent formation of the apoptosome that, in turn, triggers the activation of the caspase cascade [39]. Therefore, we next analyzed the impact of NW457 treatment +/− irradiation on the activation of the caspase cascade and caspase substrate cleavage. To this end, HCT116 cells were stimulated with 0-300 nM NW457 for 24 h, irradiated at 0-5 Gy, and 24 h later whole cell lysates were applied to Westernblot analyses of pro-caspases −9 and −3 processing as well as cleavage of the prototypical caspase substrate poly(ADP-ribose)polymerase (PARP, Figure 3A). Administration of NW457 dose-dependently stimulated the processing of pro-caspases-9 and −3 with concomitant PARP cleavage. This was further augmented by additional irradiation, most pronounced at 300 nM plus 5 Gy. To complement the proteolytic processing data, caspase activity measurements were performed using the fluorogenic peptide Ac-DEVD-AMC (N-acetyl-Asp-Glu-Val-Asp-7-amino-4-methylcoumarin), which mainly reflects the consensus cleavage motif of caspases-3 and −7 (Figure 3B). Here, a dose-dependent increase in DEVDase activity was detected particularly in the concentration range > 50 nM NW457, and this was further amplified by additional irradiation. Time course analyses of DEVDase activity and PARP cleavage disclosed that the onset of caspase activation occurred approximately 12 h after irradiation (Figure 3C, 3D). Consistent with the DNA fragmentation FACS data (Figure 2), DEVDase activity was significantly more pronounced in response to the combined treatment than after irradiation or NW457 treatment alone. These data provide first mechanistic insights that the novel HSP90 inhibitor NW457 exerts its radiosensitizing potential in colorectal cancer cells by sensitizing them for apoptosis and caspase activation *via* the intrinsic death pathway.

### Apoptosis induction and transition into secondary necrosis upon NW457 treatment in combination with irradiation relies only in part on functional p53

**Figure 4:**
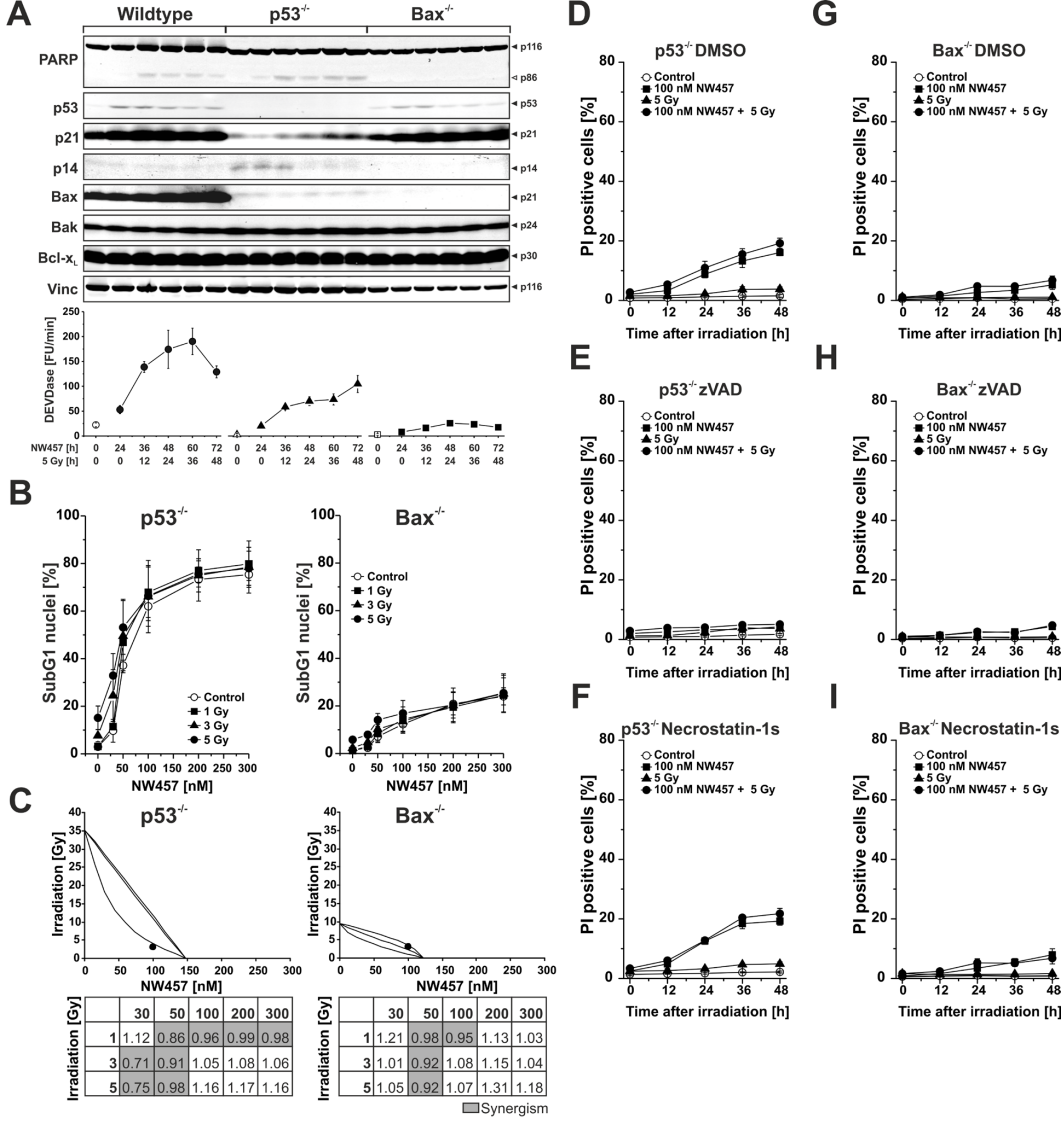
Apoptosis induction and transition into secondary necrosis upon NW457 treatment in combination with irradiation relies only in part on functional p53 HCT116 wildtype cells and p53-deficient or Bax-deficient subclones were treated with 0-300 nM NW457, irradiated at 0-5 Gy, and subjected to Westernblot analyses, DEVDase activity measurements, and FACS analyses of subG1 nuclei. **A.** *Upper panel*: Westernblot analysis of crucial signaling proteins involved in apoptosis induction after stimulation with 100 nM NW457 and 5 Gy 0-48 h after irradiation (150 μg protein per lane). Vinculin was used as loading control (10 μg protein per lane). Each blot is representative of three independent experiments. *Lower panel*: Analysis of DEVDase activity. Means ± s.d. of three experiments are shown. **B.** FACS analysis of subG1 nuclei in p53^−/−^ and Bax^−/−^ cells 48 h after irradiation +/− NW457 treatment. Means ± s.d. of three independent experiments are shown. See Figure 2B for comparison with HCT116 wildtype cells. **C.** Isobologram analyses (combination 100 nM NW457 + 3 Gy) and CI matrices of the data shown in (B). For p53^−/−^ cells, the data point of the combined treatment lies below the surface of additivity showing a synergistic mode of action. For Bax^−/−^ cells, the data point lies within the surface of additivity reflecting additive, but not synergistic behavior. CI values highlighted in grey demonstrate a synergistic mode of action between NW457 treatment and ionizing radiation (CI < 1). See Figure 2C for comparison with HCT116 wildtype cells. **D.**-**I.** Kinetics of plasma membrane disintegration measured by PI exclusion FACS staining 0-48 h after irradiation at 5 Gy +/− treatment with 100 nM NW457 in the presence or absence of the poly-caspase inhibitor zVAD-fmk or the RIP kinase-1 inhibitor necrostatin-1s. See Figure 2E-G for comparison with HCT116 wildtype cells.

Stimulation of the intrinsic apoptosis pathway by ionizing irradiation has been shown to essentially rely on p53-mediated induction of pro-apoptotic proteins, including the pro-apoptotic Bcl-2 family member Bcl-2-associated X protein (Bax) [40]. To clarify whether this also applies to NW457-mediated apoptosis induction and radiosensitization, two previously described HCT116 subclones were employed which are deficient in p53 or Bax, respectively [41, 42]. The cells were incubated with 100 nM NW457 for 24 h, irradiated at 5 Gy, and 0-48 h after irradiation whole cell lysates were subjected to Westernblot analyses as well as DEVDase activity measurements (Figure 4A). As expected, p53^−/−^ cells revealed no measurable p53 expression, and irradiation-induced upregulation of the cyclin-dependent kinase inhibitor 1 (p21^CIP1/WAF1^), a prototypical downstream target of p53, was strongly attenuated. Nevertheless, PARP cleavage and DEVDase activity were clearly detectable, although to a slightly lesser extent than in the wildtype cells. In strong contrast, genetic ablation of Bax critically interfered with caspase activation in response to NW457 treatment and irradiation, since only marginal DEVDase activity and almost no PARP cleavage were detected. These data suggest that activation of the caspase cascade in response to the combined treatment with NW457 and ionizing irradiation does only in part require functional p53 but involves Bax-dependent mechanisms of the intrinsic death pathway. The question that arises at this point is how caspase activation can be initiated in p53^−/−^ cells, which fail to induce Bax expression. Notably, there were no appreciable changes in the protein levels of Bcl-2 homologous antagonist/killer (Bak), a close relative of Bax which is discussed to be able to compensate for the loss of Bax under certain conditions, or B-cell lymphoma-extra large protein (Bcl-x_L_), an anti-apoptotic member of the Bcl-2 family, respectively [43]. However, a constitutive upregulation of the pro-apoptotic cell cycle regulator p14^ARF^ was observed in p53^−/−^ cells that might account for caspase activation in response to NW457 treatment and irradiation (Figure 4A). In this regard, it has been reported that the tumor suppressor p14^ARF^ can mediate apoptosis in a Bax- and p53-independent manner [44]. However, the downstream effectors, which couple p14^ARF^ to the signaling pathways of apoptosis, remain elusive, and other apoptosis regulators, which have not been investigated here, might also be involved.

In the next step, apoptosis induction was examined in p53- and Bax-deficient HCT116 cells on the basis of subG1 nuclei formation. Cells were treated with 0-300 nM NW457 for 24 h, irradiated at 0-5 Gy, and 48 h after irradiation FACS analyses of hypodiploid nuclei were performed (Figure 4B). p53^−/−^ cells revealed a dose-dependent increase in the percentage of subG1 nuclei comparable to the one observed in the wildtype cells, but enhancement of NW457-mediated apoptosis induction upon additional irradiation was less pronounced. Consistently, the extent of synergism between NW457 and irradiation was decreased, and the number of synergistic combinations was clearly reduced (Figure 4C). In Bax^−/−^ cells the formation of subG1 nuclei upon NW457 treatment and irradiation was very limited. Moreover, the degree of synergism between NW457 and irradiation was further reduced (Figure 4C). These findings are in line with the caspase activation data and suggest that Bax-dependent and -independent mechanisms are involved in NW457-mediated apoptosis induction and radiosensitization, whereas p53 appears largely dispensable. As compared to HCT116 wildtype cells (Figure 2E-2G), the p53^−/−^ subclone revealed a strongly reduced transit into secondary necrosis, and in the Bax^−/−^ cells secondary necrosis was barely detectable - obviously due to the limited degree of preceding apoptosis (Figure 4D, 4E, 4G, 4H). Again, no evident contribution of necroptosis was observed when necrostatin-1s was employed (Figure 4F, 4I) [35]. Westernblot analyses of mixed lineage kinase domain-like protein (MLKL) phosphorylation further strengthened this conclusion and revealed that also autophagic mechanisms as assessed by LC3A conversion, are - if at all - of minor importance in the context of NW457-mediated radiosensitization (Suppl. Figure 3) [35]. These observations are in line with other studies which have shown that crucial regulators of necroptosis and autophagy are HSP90 client proteins whose degradation upon HSP90 inhibition interferes with the coordinate execution of these processes [45–47].

### NW457 enhances irradiation-induced clonogenic cell death accompanied by degradation of selective DNA damage repair mediators and decelerated DNA damage repair

**Figure 5:**
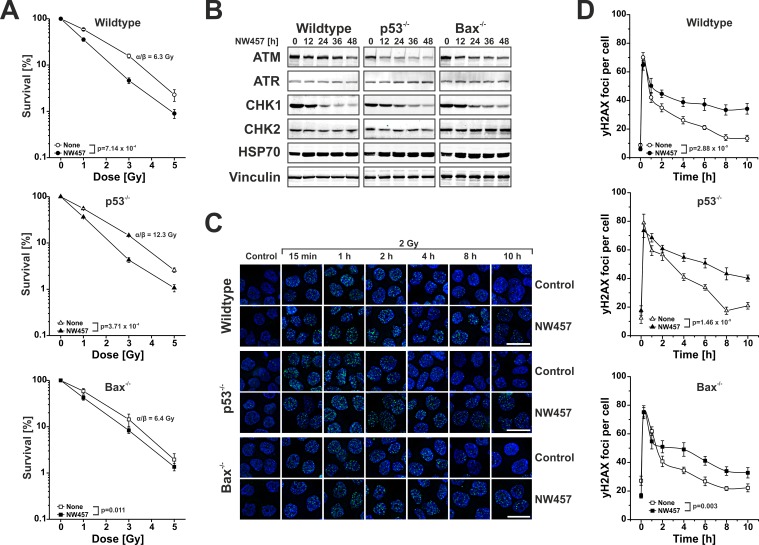
NW457 enhances irradiation-induced clonogenic cell death accompanied by degradation of selective DNA damage repair mediators and decelerated DNA damage repair HCT116 wildtype cells and p53-deficient or Bax-deficient subclones were treated with 0-30 nM NW457 and subjected to clonogenic survival assays, Westernblotting against selected DNA damage regulators, and immunofluorescence microscopy for visualization of γH2AX DNA damage repair foci. **A.** Clonogenic survival. HCT116 wildtype cells, p53^−/−^, and Bax^−/−^ subclones were subjected to colony formation assays upon irradiation at 0-5 Gy +/− pretreatment with 10 nM NW457 for 24 h. After irradiation, cells were incubated for 14 days, the numbers of colonies with more than 50 cells were counted, and the surviving fractions were calculated by normalization on the not irradiated controls (100%). Means ± s.d. of three independent experiments are depicted. α/β-values were calculated by linear-quadratic fitting, and overall comparison of the curves was performed by two-way ANOVA. **B.** Westernblot analyses of selected DNA damage response regulators. Cells were treated with 20 nM NW457 for 0-48 h, and whole cell protein extracts were subjected to 4-8% or 6-15% SDS-PAGE with subsequent immunoblotting against ATM, ATR, CHK1, and CHK2 (300 μg protein per lane). Vinculin served as a loading control (30 μg protein per lane), and HSP70 (30 μg protein per lane) is depicted to show induction of the heat shock response. **C.** Immunofluorescence microscopy of γH2AX DNA damage repair foci. Cells were pretreated +/− 30 nM NW457 for 24 h and irradiated at 2 Gy. Immunofluorescence staining for γH2AX DNA damage repair foci (green) was performed at the indicated times. Nuclei were visualized by Hoechst 33342 staining. Scale bars depict 20 μm. **D.** Quantification of γH2AX DNA damage repair foci staining as shown in (C). 20 nuclei per condition were analyzed, and the numbers of γH2AX foci per cell are depicted as means ± s.e.m. Overall comparison of curves was performed by two-way ANOVA.

In order to investigate the long-term effects of NW457 on irradiation-induced cell death, clonogenic survival assays were performed. This endpoint is classically considered as one of the most relevant *in vitro* endpoints to predict potential radiosensitizing effects *in vivo*. HCT116 wildtype, Bax^−/−^, and p53^−/−^ cells were seeded in varying densities, irradiated at 0-5 Gy with or without preincubation with 10 nM NW457 for 24 h, and cultivated for 14 days. Afterwards, the number of resulting colonies was counted, and the surviving fraction was determined by normalization to the number of seeded cells and the respective plating efficiency. Irradiation reduced the clonogenicity of all three cell lines in a dose-dependent manner (Figure 5A). However, the α/β-values determined by linear-quadratic fitting, suggested that the DNA damage repair capacity of p53^−/−^ cells is considerably compromised as compared to Bax^−/−^ or wildtype cells [48]. Importantly, pretreatment with NW457 enhanced irradiation-induced clonogenic cell death in all three cell lines to a strong and statistically significant extent. Hence, NW457 apparently exerts a long-term radiosensitizing effect in HCT116 cells, which is essentially independent of functional p53 and Bax. These findings are to a certain degree contrasting the apoptosis data. However, it should be noted that clonogenic survival was measured with a lower concentration of NW457 (10 nM), since no colony formation could be detected in the presence of 100 nM NW457. In order to elucidate in greater depth, which mode of cell death underlies NW457-mediated radiosensitization in this setup, cell death analyses were performed with a reduced concentration of NW457 (10 nM) until day 6 and under the conditions with limiting cell numbers that were used for clonogenic survival assays until day 14. We observed that irradiation alone stimulated a delayed onset of apoptosis around day 4, which was significantly enhanced by additional HSP90 inhibition and virtually independent of the p53 and/or Bax status. As was the case for higher NW457 concentrations, apoptotic cells transited into post-apoptotic, secondary necrosis later on. Induction of senescence was also detected. Here however, no differences between irradiation alone and the combined treatment were noticed. Long-term cell death assays performed over 14 days under the conditions of the colony formation assays revealed apoptosis and senescence to be the major mechanisms underlying the abrogation of clonogenicity upon the combined treatment with NW457 and ionizing irradiation, and no significant differences between the HCT116 subclones were observed (Suppl. Figure 4).

Clonogenic cell death in response to irradiation is commonly considered to derive from lethal DNA damage. Therefore, we next examined how HSP90 inhibition by NW457 affects different regulators of the DNA damage response and the kinetics of DNA damage repair. HCT116 wildtype, Bax^−/−^, and p53^−/−^ cells were treated with 20 nM NW457 for 0-48 h, and the protein levels of the proximal DNA damage response kinases ataxia telangiectasia mutated protein (ATM), ataxia telangiectasia and RAD3-related protein (ATR), checkpoint kinase 1 (CHK1), and checkpoint kinase 2 (CHK2) were analyzed by Westernblotting. For ATM and CHK1, a clear and time-dependent decrease was observed in all three cell lines (Figure 5B), which is in line with other reports showing that regulators of the DNA damage response are most susceptible to HSP90 inhibition already at very low inhibitor concentrations [11]. The degradation of the two crucial upstream kinases of the DNA damage response translated into decelerated DNA damage repair as determined by phosphorylated histone H2AX (γH2AX) staining (Figure 5C-5D). Time course analyses displayed similar appearance rates of γH2AX DNA damage repair foci in control and NW457-pretreated cells, but the clearance of γH2AX foci was significantly delayed upon preincubation with NW457, and substantial numbers of residual γH2AX foci were detected even 10 h after irradiation. This was the case for HCT116 wildtype cells as well as for the Bax^−/−^ and p53^−/−^ subclones. Taken together, these data suggest that HSP90 inhibition by NW457 involves the degradation of selective upstream regulators of the DNA damage response resulting in compromised repair of irradiation-induced DNA damage and reduced clonogenic survival, which is mainly driven by apoptotic cell death mechanisms and largely independent of functional p53 and Bax. To confirm that NW457-mediated radiosensitization is not limited to HCT116 cells and subclones derived thereof, selected experiments were also performed with human HCT8 and murine CT26 colorectal cancer cells. Synergism analyses on the basis of apoptotic subG1 nuclei measurements and clonogenic survival assays provided similar results as had been obtained with HCT116 cells (Suppl. Figure 5). These findings encouraged us to use heterotopically transplanted tumors of CT26 cells on the flanks of syngeneic, immunocompetent Balb/c mice to assess the *in vivo* performance of NW457 treatment alone or in combination with radiotherapy.

### NW457 improves irradiation-mediated tumor control *in vivo* and reveals little hepatotoxicity

**Figure 6:**
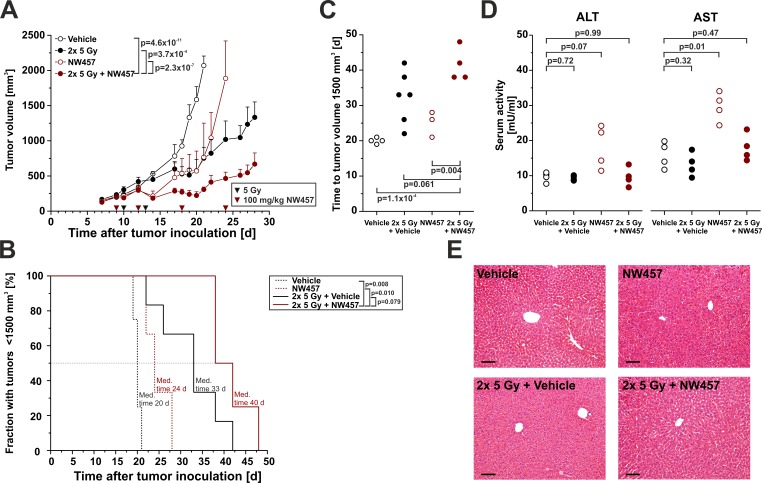
NW457 improves irradiation-mediated tumor control *in vivo* and reveals little hepatotoxicity NW457-mediated radiosensitization was assessed *in vivo* by using a syngeneic heterotopic Balb/c mouse model of CT26 colorectal tumor cells growing on the right flank. 1.2x 10^6^ CT26 cells were injected subcutaneously, and tumors were allowed to grow for 9 days. Mice with tumors of less than 300 mm^3^ were distributed into four groups and subjected to the following treatments: Vehicle only (*n* = 4), NW457 only (n = 6), 2x 5 Gy + vehicle (*n* = 6), and 2x 5 Gy + NW457 (*n* = 5). NW457 (100 mg/kg) and DMSO were administered by i.p. injection on days 9, 12, 18, and 24 (red arrowheads). Local irradiation was performed on days 10 and 13 (black arrowheads). Tumor growth and liver toxicity were monitored. **A.** Tumor growth. Tumor dimensions were measured every 1-3 days and tumor volumes were calculated using the formula (width x width x length)/2. Means + s.e.m. are shown. Overall comparison of curves was performed by two-way ANOVA. **B.** Kaplan-Meier survival curves. Tumor-specific death was scored when the tumor volume exceeded 1,700 mm^3^ and animals had to be sacrificed. Median survival times were determined, and *p*-values were calculated by log-rank test. **C.** Time to tumor volume 1,500 mm^3^. The time after which tumor size reached 1,500mm^3^ was determined, and compared by unpaired Student's *t*-test. **D.** Serum levels of liver enzymes. Selected animals were sacrificed 2 days after the second irradiation fraction, and serum was collected for ALT and AST analyses. *p*-values were calculated by unpaired Student's *t*-test. **E.** Liver histology. Livers of animals sacrificed 2 days after the second irradiation fraction were explanted, formalin-fixed, paraffin-embedded, and 4 μm sections were stained with hematoxylin-eosin. Representative images of 1 out of 3-4 animals per group are shown. Scale bars represent 100 μm.

The anti-tumor efficacy of HSP90 inhibition in combination with radiotherapy has rarely been examined *in vivo*, and data on HSP90 inhibitor-mediated radiosensitization in colorectal cancer models are scarce [49]. We therefore sought to elucidate the anti-tumor effects of NW457 in combination with radiotherapy in a heterotopic transplantation model of CT26 colorectal cancer cells on Balb/c mice. Mice were subcutaneously inoculated with murine CT26 cells on day 0, and tumors were allowed to grow for 9 days. Mice with tumors < 300 mm^3^ were randomly distributed into four groups: NW457 (4x 100 mg/kg i.p.), dimethyl sulfoxide (DMSO), 2x 5 Gy + NW457 (4x 100 mg/kg i.p.), and 2x 5 Gy + DMSO. NW457 or DMSO as vehicle control were administered intraperitoneally on days 9, 12, 18, and 24. Irradiation was carried out in two fractions of 5 Gy on days 10 and 13 after tumor implantation. Tumor volume was monitored every 1-3 days, and mice carrying tumors ≥ 1,700 mm^3^ were sacrificed in accordance with legal requirements. Exponential tumor growth was observed in the vehicle control group, and until day 21 all animals had to be sacrificed (Figure 6A, 6B). Treatment with NW457 alone resulted in an early delay in tumor growth from day 10 to day 20. Later on, tumors resumed exponential growth exceeding a mean volume of 1,700 mm^3^ until day 24. Radiotherapy plus vehicle induced a potent reduction in tumor growth, with four of six animals carrying tumors < 1,700 mm^3^ on day 27 and the average tumor volume being 1,234 ± 246 mm^3^ (mean ± s.e.m.). Most importantly, the strongest inhibitory effect on tumor growth was observed in the group that had received the combined therapy comprising 2x 5 Gy plus NW457. Here, the early delay in tumor growth, which was also observed in the NW457 only group, together with long-term growth inhibitory effects beyond day 20 resulted in an average tumor volume of 546 ± 110 mm^3^ (mean ± s.e.m.) on day 27. Compared to the radiotherapy plus vehicle group and the NW457 only group, a significant reduction in tumor growth was detected in the radiotherapy plus NW457 group.

Kaplan-Meier time-to-event analyses were performed for the tumor-specific survival of the four treatment groups (Figure 6B). The median survival of the vehicle only group was 20 days, and all animals had to be sacrificed due to tumor volumes exceeding 1,700 mm^3^ within 21 days after tumor inoculation. Mice receiving NW457 alone showed a median tumor-specific survival of 24 days. Interestingly, radiotherapy with 2x 5 Gy prolonged the median survival to 33 days. However, the strongest prolongation in survival was observed in the group which was subjected to the combined therapy with 2x 5 Gy irradiation plus NW457, as this therapeutic regime resulted in a median survival of 40 days. Log-rank tests revealed that the tumor-specific survival in the radiotherapy plus NW457 group was significantly prolonged compared to the groups receiving NW457 only (*p* = 0.010) or vehicle only (*p* = 0.008), respectively. As compared to the group receiving radiotherapy plus vehicle, the tumor-specific survival in the radiotherapy plus NW457 group was prolonged as well. However, this difference did yet not reach statistical significance (*p* = 0.079). Basically the same results were obtained when analyzing the time to tumor volume = 1,500 mm^3^ (Figure 6C).

Finally, we addressed the issue of *in vivo* hepatotoxicity. Randomly selected animals were sacrificed 2 days after the second irradiation fraction when the therapeutic stress was considered to be maximal. Serum levels of the liver enzymes alanine aminotransferase (ALT) and aspartate aminotransferase (AST) were measured, and sections of formalin-fixed, paraffin-embedded liver samples underwent routine histopathological examination. Here, mice of all experimental groups displayed a normal histomorphology of liver parenchyma without evident signs of toxic alterations (Figure 6E), although slight, but statistically significant increases in ALT and AST serum levels were detected in the NW457 only group (Figure 6D).

In summary, our findings identify the novel HSP90 inhibitor NW457 as a promising candidate for combined modality approaches together with radiotherapy. It potently radiosensitizes colorectal cancer cells irrespective of functional p53 or Bax *in vitro* and shows encouraging anti-tumor efficacy accompanied by very little hepatotoxicity in combination with radiotherapy *in vivo.*

## DISCUSSION

HSP90 has emerged as a promising target for anti-cancer therapy, and currently 17 different pharmacological inhibitors are undergoing clinical testing [10] (http://clinicaltrials.gov). Of vital importance for the clinical success of HSP90 inhibitors is the therapeutic window, which clearly depends on their selectivity for tumor cells over non-malignant cells and their overall tolerability. Tumor cells were repeatedly shown to be more sensitive to HSP90 inhibitors than non-transformed cells, and non-toxic inhibitor concentrations demonstrated potent anti-tumor activity *in vivo* [50–52]. Different mechanisms are currently discussed to underlie this tumor specificity. Firstly, cellular malignancy is driven by mutant and/or overexpressed oncoproteins, which stringently require HSP90 chaperoning for correct folding, stability, and activity [2]. Secondly, due to their aneuploidy tumor cells frequently suffer from proteotoxic stress, and the tumor microenvironment is characterized by nutrient deprivation, acidosis, and hypoxia. In consequence, the heat shock response is activated, and HSP90 enables tumor cells to survive and proliferate in this harsh milieu [2]. Thirdly, HSP90 in tumor cells is considered to be predominantly engaged in multi-chaperone complexes which exhibit higher affinity for HSP90 inhibitors and thus render ‘normal’ HSP90 complexes in non-malignant cells basically unaffected [52]. Finally, tumor cells are known to expose HSP90 on their cell surface, and this ectopic HSP90 can contribute to an increase in cellular uptake rates and thus the specific accumulation of HSP90 inhibitors in tumor cells [53].

Despite these encouraging tumor-specific effects, the clinical translation of HSP90 inhibitors has been largely impeded by their suboptimal pharmacological properties, including poor water solubility and bioavailability, as well as substantial hepatotoxicity [3]. The quinone moiety of geldanamycin and its derivatives 17-AAG and 17-DMAG is metabolized by members of the NADPH-cytochrome P450 reductase and NADH cytochrome b5 reductase family, generating semiquinones and superoxide radicals [54]. Additionally, quinones react with thiol groups and form toxic conjugates [31]. The respective reductases and thiols are essentially involved in xenobiotic metabolism and thus are enriched in the liver. Accordingly, the toxicity of geldanamycin-derived HSP90 inhibitors becomes preferentially evident in this organ. In contrast, radicicol and its derivatives are not based on a quinone structure, hence avoiding unwanted biotransformation responsible for hepatotoxicity [22, 23, 55]. However, the *in vivo* availability of radicicol turned out to be very limited [21, 56].

In the present study, we made use of the novel HSP90 inhibitor NW457, a radicicol derivative of the pochoxime family, which was developed with the motivation to increase water solubility and bioavailability, while reducing hepatotoxicity at the same time [20–23]. Potent HSP90 inhibition was observed in human colorectal cancer cells upon treatment with nanomolar concentrations of NW457 as measured by the degradation of prototypical HSP90 client proteins. In parallel, NW457 was well tolerated by primary murine hepatocytes *in vitro* and exhibited only limited hepatotoxicity *in vivo*.

We utilized HSP90 inhibition by NW457 in combination with ionizing irradiation, since radiotherapy represents a central treatment modality for cancers of the rectum - implemented either as neoadjuvant or adjuvant therapy alone or in combination with chemotherapy, respectively [24–26]. For colon cancer however, radiotherapy is only occasionally used on a case-by-case basis owing to the high degree of mobility of the colon and the resultant side effects for the normal tissue if irradiation volumes with larger safety margins were administered. Substances which preferentially radiosensitize the tumor tissue in principal should help to widen the therapeutic index in this regard. From a molecular perspective, HSP90 inhibition appears as a particularly promising partner for radiotherapy, since regulators of the DNA damage response have been reported to be most susceptible to HSP90 inhibition among the plethora of HSP90 client proteins [11]. Indeed, treatment of colorectal cancer cells with NW457 resulted in rapid degradation of ATM and CHK1, two essential upstream kinases of the DNA damage response. This is in accordance with findings of other studies, which showed that targeting HSP90 with small-molecule inhibitors induces the destabilization of different DNA damage response regulators, including ATM, CHK1, DNA-dependent protein kinase (DNA-PK), DNA repair protein RAD51 homologue 1 (RAD51), breast cancer 1 (BRCA1), and others [12, 13, 15, 19, 49, 57, 58]. Importantly, homologous recombination and non-homologous end joining, the two major mechanisms which operate to repair irradiation-induced DNA double strand breaks, appear to be affected to comparable extents [12, 13, 49, 59, 60].

The disappearance of DNA damage regulators translated into delayed DNA damage repair and reduced clonogenic survival. Similar results have been reported for other HSP90 inhibitors in other cell systems [13, 15, 59]. Intriguingly, the impact of NW457 on DNA damage repair and clonogenic survival in our study was virtually independent of the cellular p53 and Bax status. In accordance, other groups also described that p53 is dispensable for HSP90 inhibitor-induced clonogenic cell death [19, 61]. Potential underlying mechanisms, including an abrogation of p53-dependent irradiation-induced G2-arrest and premature mitotic entry resulting in mitotic catastrophe, have been proposed [61]. Nevertheless, p53′s role in HSP90 inhibitor-mediated radiosensitization is controversially discussed, and further in-depth analyses are needed in order to clarify whether cells with mutant and/or lacking p53 function are more or less susceptible to HSP90 inhibitor-dependent radiosensitization than their wildtype counterparts [13, 61–63].

Limited p53 dependence was also observed in our mechanistic studies on apoptosis induction. In a largely synergistic manner, NW457 treatment and ionizing irradiation stimulated caspase activation, caspase substrate cleavage, apoptotic chromatin condensation, and DNA fragmentation. Upon treatment with higher concentrations of NW457, apoptosis was predominantly triggered *via* the intrinsic death pathway, as the mentioned characteristics were strongly attenuated in Bax-deficient cells. However, for apoptosis induction by lower concentrations of NW457 in combination with irradiation, also Bax appeared to be dispensable. Induction of apoptotic cell death upon HSP90 inhibition has been reported for other compounds, and it has been shown to be associated with caspase activation, PARP cleavage, and release of mitochondrial cytochrome c [15, 18, 60, 64]. p53 was not necessarily required in this regard, since caspase activation, caspase substrate cleavage, and the formation of hypodiploid nuclei were well observed in p53-deficient cells in response to NW457 treatment and irradiation. Given that p53 is mutated or lost in up to 50% of colorectal cancers [65], these findings are of specific clinical relevance and suggest that NW457-based therapies may also be efficient in tumors lacking functional p53. On the molecular level, several concepts on how p53-independent apoptosis in response to DNA damage is stimulated are being discussed. One of these involves the pro-apoptotic cell cycle regulator p14^ARF^, which in our study was shown to be constitutively upregulated in p53-deficient cells and which was previously reported to induce intrinsic apoptosis in a Bak-dependent manner [44]. Additionally, p53-independent apoptosis induction can be mediated by the p53 relatives p63 and p73. Both exhibit p53-like properties and are able to transactivate typical p53-responsive genes, including p21^CIP/WAF^, Bax, phorbol-12-myristate-13-acetate-induced protein 1 (Noxa), and p53-upregulated modulator of apoptosis (Puma), finally leading to cell cycle arrest and/or programmed cell death [66]. Of note, p73-dependent apoptosis induction was repeatedly described in tumor cells lacking functional p53 and may thus also be operative in p53-deficient HCT116 cells upon NW457 treatment and irradiation [67].

In our analyses, Bax^−/−^ cells revealed a profound reduction in apoptosis induction upon treatment with 100 nM NW457 alone or in combination with irradiation, whereas apoptosis induction and enforcement of clonogenic cell death upon treatment with 10 nM NW457 followed by irradiation were basically independent of functional Bax. Obviously, different concentrations of NW457 can trigger different apoptotic mechanisms. This is reflected not only by the differences in Bax dependency but also by the different kinetics.

The *in vivo* anti-tumor efficacy of HSP90 inhibition as monotreatment has been proven by several studies [9, 50, 60, 68]. However, only limited data are available for combined approaches together with radiotherapy [16–19]. Despite different pharmacological compounds, dose and radiation schedules, all studies clearly showed that the therapeutic effect as measured by a delay in tumor growth and a prolongation of survival was superior to that of the monotherapies. To the best of our knowledge, this is the first study which combines HSP90 inhibition with radiotherapy in a syngeneic colorectal cancer model. NW457 treatment alone resulted only in a marginal and temporary inhibition of tumor growth, whereas radiotherapy alone was slightly more effective. Importantly, the administration of NW457 plus radiotherapy led to a significant delay in tumor growth as compared to both single agent treatments. It should be noted that the CT26 colorectal cancer model is p53-proficient but carries - as HCT116 cells do - an activating mutation in kirsten rat sarcoma viral oncogene homologue (KRAS), an oncogene which is of pivotal importance in colorectal cancer, as mutations have been reported in approximately 50% of all cases [69]. This again, underlines the clinical relevance of our findings. However, if and to which extent Bax-dependent mechanisms of intrinsic apoptosis are involved in NW457-mediated radiosensitization *in vivo* needs further clarification - preferentially by using genetically engineered mouse models.

Coming back to the original motivation that NW457 was developed with the aim of improving water solubility and bioavailability while reducing hepatotoxicity [22, 23], it can be said that on the level of light-microscopy, there was no clear evidence of toxic alterations of liver histomorphology in NW457-treated mice (100 mg/kg), and serum levels of ALT and AST were only slightly increased (< 25 mU/ml for ALT, < 35 mU/ml for AST). These results confirm the findings of our *in vitro* analyses, although comprehensive toxicopathological analyses of *in vivo* hepatotoxicity were not performed, and were not in the scope of the present study. For GA, serum levels of ALT and AST were reported to exceed 500 mU/ml when administered at 15 mg/kg [70]. Even for the toxicology-optimized GA derivatives 17-AAG and 17-DMAG (15 mg/kg), the resulting ALT and AST serum levels were clearly higher (> 25 mU/ml for ALT, > 45 mU/ml for AST) than the ones that were observed for NW457 administration at 100 mg/kg in our study [70, 71].

In summary, targeting HSP90 with the novel radicicol-derived pochoxime family member NW457 appears as a promising approach of radiosensitization with good *in vivo* efficacy and tolerability.

## MATERIALS AND METHODS

### Cells and reagents

Primary hepatocytes were isolated from adult C57BL/6 mice by liver perfusion and percoll density centrifugation (approved by the *Regierung von Oberbayern*). Briefly, mice were anaesthetized with isoflurane (Abbott Laboratories, Ludwigshafen, Germany) and the peritoneal cavity was opened. A 20 G needle (30 mm length) was introduced into the vena porta, the vena cava was immediately opened, and the liver was perfused with prewarmed perfusion medium (0.9% NaCl, 0.05% KCl, 0.2% HEPES, 0.008% EDTA, pH7.4) using a peristaltic pump (5 ml/min) until drained of blood. Liver digestion was performed by adding 33 μg/ml Liberase^TM^ collagenase (Roche applied Science, Penzberg, Germany) to the perfusion medium, and perfusion was maintained until the liver felt soft (5-6 min). The perfused liver was explanted from the peritoneal cavity, the gall bladder was removed, and the liver was carefully disrupted using two Q-Tips. The cell suspension spreading out of the digested liver was forced through a 100 μm cell strainer (BD Biosciences, Heidelberg, Germany) and diluted with William's E complete medium (William's E medium supplemented with 4% heat-inactivated fetal calf serum (FCS) (both from Life technologies, Karlsruhe, Germany) 100 U/ml penicillin, and 0.1 mg/ml streptomycin (Lonza, Basel, Switzerland). Hepatocytes were sedimented at 35 g for 5 min and the cell pellet was resuspended in William's E complete medium. Separation of parenchymal, non-parenchymal, and dead cells was performed using percoll gradient centrifugation with three densities (1.12 g/ml, 1.08 g/ml, and 1.06 g/ml Easycoll^TM^, Biochrom, Berlin, Germany) at 750 g for 20 min. Subsequently, the two upper gradient layers containing cell debris and non-parenchymal cells were carefully removed and viable hepatocytes in the lowest layer were collected. Hepatocytes were washed in William's E complete medium, seeded into gelatin-coated dishes and cultured in William's E complete medium supplemented with 50 ng/ml epidermal growth factor (EGF), 1 μg/ml insulin, 10 μg/ml transferrin, and 1.3 μg/ml hydrocortisone (all from Sigma-Aldrich, Taufkirchen, Germany).

The human colorectal cancer cell line HCT116 and its p53^−/−^ and Bax^−/−^ subclones were generously provided by Bert Vogelstein [42, 72]. Cells were cultured in McCoy's 5A medium (Life Technologies) supplemented with 10% heat-inactivated FCS, 100 U/ml penicillin, and 0.1 mg/ml streptomycin at 37°C and 7.5% CO_2_. Human HCT8 and mouse CT26 cells derived from Balb/c mice were obtained from ATCC and cultured in DMEM or RPMI 1640 medium (Life Technologies) respectively, supplemented with 10% heat-inactivated FCS, 100 U/ml penicillin, and 0.1 mg/ml streptomycin at 37°C and 5% CO_2_. Identity of cell lines was confirmed by short tandem repeat (STR) typing (service provided by the DSMZ, Brunschweig, Germany).

The novel HSP90 inhibitor NW457 (*epi*-pochoxime F) was synthesized and purified as described before [20–23], and GA was obtained from Merck Millipore (Darmstadt, Germany). Both inhibitors were stored as 10 mM stock solutions in DMSO at −20°C and were freshly diluted in the appropriate culture medium immediately before use. The respective DMSO concentrations were used as vehicle control. The poly-caspase inhibitor zVAD-fmk was purchased from Bachem (Bubendorf, Switzerland), necrostatin-1s from Biovision (Milpitas, CA, USA), staurosporine from Sigma-Aldrich, tumor necrosis factor (TNF) from R&D Systems (Wiesbaden, Germany), cycloheximide (CHX) from Merck Millipore, and Rapamycin from LC Laboratories (Woburn, MA, USA). All other reagents were obtained from Sigma-Aldrich, if not stated otherwise.

### X-ray treatment

Cells were irradiated at the indicated doses with a Mueller RT-250 γ-ray tube (200 kV and 10 mA, Thoraeus filter, 1 Gy in 1 min 52 s) or an RS-225 cabinet (200 kV and 10 mA, Thoraeus filter, 1 Gy in 1 min 3 s; Xstrahl, Camberley, UK) as described previously [73].

### Immunofluorescence microscopy

Fluorescence microscopy was performed using an inverse epifluorescence microscope (Zeiss AxioObserver Z1) equipped with a Zeiss Plan-Neofluar 63x/1.3 glycerol objective, AxioVision 4.8 software, and an AxioCam Mr3 camera (Carl Zeiss, Jena, Germany). The following filters were used: BP 365/12 for Hoechst 33342, BP 470/40 for FITC, and BP 550/25 for Alexa Fluor 568 (all from Carl Zeiss).

For examination of hepatocyte morphology, 8x 10^3^ primary mouse hepatocytes per well were seeded into Ibidi 8-well μ-slides (Ibidi, Martinsried, Germany) coated with 0.2% gelatin. Upon adherence for 16 h, cells were treated with the indicated concentrations of NW457, GA, or DMSO as vehicle control for 24 h. Afterwards the medium was removed, cells were washed in phosphate-buffered saline (PBS), and fixed in 3.7% isotonic paraformaldehyde (Merck Millipore) containing 0.1% Triton X-100 for 10 min at room temperature. Hepatocytes were washed in PBS and permeabilized in 0.5% isotonic Triton X-100 for 5 min. After blocking of unspecific binding sites in 3% isotonic bovine serum albumin (BSA) + 0.1% Triton X-100 for 1 h, cells were stained with Alexa Fluor 568-labeled phalloidin (Life Technologies) and FITC-labeled monoclonal anti-β-tubulin antibody (Sigma-Aldrich) for 2 h at room temperature. Cells were washed in PBS + 0.1% Triton X-100, and stained with Hoechst 33342 (2 μg/ml) for 10 min. Finally, hepatocytes were washed twice in PBS + 0.1% Triton X-100, and mounted in Fluoromount medium (Sigma-Aldrich).

To analyze the kinetics of DNA damage repair in the presence or absence of NW457 cells were stained for γH2AX as described previously [57]. Briefly, 2.5x 10^5^ HCT116 cells were seeded into 24-well plates supplemented with coverslips. After adherence for 16 h, cells were treated with 30 nM NW457 or DMSO for 24 h and subsequently irradiated with 2 Gy. Not irradiated cells served as controls. At the indicated time points, cells were washed in PBS and fixed in 3.7% isotonic paraformaldehyde (containing 0.1% Triton X-100) for 10 min. Cells were permeabilized in 0.5% isotonic Triton X-100 for 5 min, unspecific binding sites were blocked in 3% isotonic BSA + 0.1% Triton X-100 for 1 h, and cells were stained with anti-γH2AX-antibody (Merck Millipore) for 2 h at room temperature. After washing in PBS + 0.1% Triton X-100 cells were stained with Alexa Fluor 488-labeled anti-mouse-IgG antibody (Life Technologies) for 45 min and with Hoechst 33342 (2 μg/ml) for 10 min. Cells were washed in PBS + 0.1% Triton X-100 and mounted onto glass slides (Thermo Scientific, Schwerte, Germany). 25 Z-stacks with 250 nm distance were captured and subsequent deconvolution was performed using the AxioVision 4.8 software.

Microscopic quantification of apoptotic cells was performed by staining with Hoechst 33342. 5x 10^5^ cells per well were seeded into 24-well plates and treated as indicated. Afterwards, nuclei were directly stained in the culture dishes with 3 μg/ml Hoechst 33342 for 15 min, and examined by fluorescence microscopy. Cells revealing features of chromatin condensation (patchy Hoechst staining) or nuclear fragmentation were considered apoptotic. For each condition, at least 400 nuclei were counted in randomly selected microscopic fields of two independent wells, and the percentage of apoptotic cells was calculated.

### Viability tests

Cell viability was determined by using the Alamar Blue reagent (BioRad, Munich, Germany) according to the manufacturer's recommendations. Briefly, 1x 10^4^ cells per well were seeded into 96-well plates and allowed to adhere overnight. Cells were treated as described and incubated for the indicated times. Subsequently, the medium was removed and cells were washed once in culture medium. Alamar Blue reagent was added at 1/10 volume of culture medium and resazurin reduction was allowed for 4-7 h at 37°C. Resorufin fluorescence was measured using a Synergy Mx microplate reader (BioTek, Bad Friedrichshall, Germany). Results were calibrated on the vehicle-treated controls (100% viability).

### Colony formation assays

Clonogenic survival was examined in colony formation assays. Cells were seeded as single cell suspensions into 6-well plates in a range of 100-600,000 cells per well in order to yield 40-80 colonies per well depending on the different stimuli. After adherence for 4 h, cells were irradiated immediately, or treated with 10 nM NW457 for 24 h. The drug-containing medium was replaced by drug-free medium, cells were irradiated at the indicated doses, and colony formation was allowed for the following 14 days. Subsequently, cells were fixed and stained in 80% ethanol containing 0.3% methylene blue, and colonies with more than 50 cells were counted. The percentage of surviving cells was normalized on the corresponding plating efficiency (survival at 0 Gy after pretreatment with DMSO or NW457, respectively).

### HSP70 ELISA

HSP70 release into culture supernatants was monitored by using an HSP70 ELISA kit (DuoSet, R&D Systems) according to the manufacturer's instructions. In 6-well plates, 0.5-1x 10^6^ HCT116 cells per well were treated as indicated, and cell-free culture supernatants were harvested. 100 μl of the 1:5 diluted supernatants were incubated in 96-well plates precoated with mouse anti-human HSP70 capture antibody for 2 h. Plates were washed, and a biotinylated rabbit anti-human HSP70 detection antibody was added for 2 h. Plates were again washed and incubated with streptavidin-horseradish peroxidase for 20 min. After extensive washing, the assay was developed with tetramethylbenzidine substrate solution for 20 min, and the optical density was measured at 450 nm in a microplate reader (Synergy Mx, BioTek). Concentrations of released HSP70 were calculated using a standard curve prepared from a dilution series of recombinant human HSP70.

### SDS-PAGE and Westernblot analyses

Reducing gradient SDS-PAGE and Westernblot analyses of whole cell lysates (20-300 μg total protein per lane) were performed as described previously [74]. Whole cell lysates were prepared in lysis buffer (50 mM Tris pH 7.6, 150 mM NaCl, 1% Triton X-100, 3 μg/ml aprotinin, 3 μg/ml leupeptin, 3 μg/ml pepstatin, and 2 mM phenylmethylsulfonyl fluoride), boiled in Laemmli buffer and subjected to electrophoretic separation. Afterwards, proteins were transferred to PVDF Immobilon FL membranes (Merck Millipore). Membranes were blocked with 5% low-fat milk in TBST buffer (13 mM Tris-HCl pH 7.5, 150 mM NaCl, and 0.02% Triton X-100) and incubated with different primary antibodies. The following antibodies were used: anti-PARP (Trevigen Biozol, Eching, Germany), anti-caspase-9 (R&D Systems), anti-caspase-3 full length, anti-BRAF, anti-HSP70, anti-CHK2 (all from BD Biosciences), anti-CHK1, anti-vinculin (Sigma-Aldrich), anti-cleaved caspase-3, anti-Bax, anti-Bak, anti-Bcl-x_L_, anti-HSP90, anti-p14^ARF^, anti-p21^CIP1/WAF1^, anti-p53, anti-phospho-MLKL (T357/S358), anti-LC3A (all from Cell Signaling Technology, Leiden, The Netherlands), anti-ATM, and anti-ATR (Merck Millipore). After incubation with the corresponding IRDye-conjugated secondary antibodies (LI-COR Biosciences, Bad Homburg, Germany) and extensive washing in TBST buffer, IRDye fluorescence was measured with a LI-COR Odyssey scanner.

### Caspase activity tests

Effector caspase activity was examined in an enzymatic assay with whole cell protein extracts and the fluorogenic peptide Ac-DEVD-AMC (Bachem, Bubendorf, Switzerland) [75]. Briefly, whole cell protein extracts were prepared as described for Westernblotting, and 10 μg of total protein extracts were incubated for 1 h at 37°C with 50 μM Ac-DEVD-AMC in 200 μl of buffer containing 50 mM HEPES pH 7.3, 100 mM NaCl, 10% sucrose, 0.1% CHAPS, and 10 mM dithiothreitol. The kinetics of AMC release was measured spectrofluorimetrically in a microplate reader (Synergy Mx, BioTek, Excitation 360/9 nm, Emission 460/9 nm). Relative caspase activity was determined from the slope of the resulting linear regressions, and is expressed in arbitrary fluorescence units per minute.

### Flow cytometric measurement of surface antigen expression, apoptotic DNA fragmentation, PI exclusion, senescence-associated β-galactosidase activity, and cell cycle distribution

For all FACS measurements an LSRII cytometer (BD Biosciences) was employed, and data were analyzed with FACSDiva (BD Biosciences) or FlowJo 7.6.5 software (Tree Star Inc., Ashland, OR, USA), respectively. The following FACS antibodies were used: anti-EGFR-PE, anti-EPHA2-FITC (both from BD Biosciences), and anti-HSP70-FITC (Multimmune, Munich, Germany).

For surface marker staining, 1x 10^5^ cells were incubated with 2 μl of the directly coupled antibodies or the matching isotype controls in 50 μl FACS staining buffer (BD Biosciences) for 30 min on ice. After two washing steps in FACS staining buffer, cells were analyzed by flow cytometry. Relative surface expression was calculated as the median fluorescence intensities of the antibody-stained samples divided by the corresponding isotype controls. In case of HSP70 surface staining, additional PI exclusion staining was performed with 2 μg/ml PI, and the mean fluorescence intensities of the antibody-stained samples or the matching isotype controls are displayed, respectively.

Staining of apoptotic DNA fragmentation was performed as described before [75]. Briefly, in 96-well plates, 5-6x 10^3^ cells per well were treated with NW457 and irradiated as indicated. Subsequently, culture plates were spun down and supernatants were removed. Nuclei were released and stained for their DNA content by incubating the cells with hypotonic PI staining buffer (50 μg/ml PI, 0.1% (w/v) tri-sodium citrate dihydrate, 0.1% (v/v) Triton X-100) at 37°C for 5 min. Forward scatter (FSC), sideward scatter (SSC), and PI fluorescence were analyzed. Doublet exclusion was performed, and all nuclei with less than diploid DNA content were considered apoptotic.

Plasma membrane integrity was assessed by flow cytometric PI exclusion staining. Cells were collected by trypsinization, resuspended in PBS with 5 μg/ml PI, and subjected to FACS analyses. Cells with positive PI staining were considered necrotic.

Cellular senescence was detected by using a FACS-based activity test for senescence-associated β-galactosidase with the fluorogenic substrate 5-dodecanoylaminofluorescein-di-β-galactopyranoside (C12-FDG, Life Technologies) as described previously [57]. Briefly, following lysosomal alkalinization with 100 nM bafilomycin A1 (Tocris R&D Systems, Heidelberg, Germany), 50 μM C12-FDG was added, and substrate conversion was allowed for 1 h at 37°C. After washing in PBS, cells were collected by trypsinization and subjected to FACS analyses. Cells with high C12-FDG and high SSC signal were considered senescent.

FACS-based cell cycle analyses were performed by staining ethanol-fixed cells with anti-phospho-histone H3 antibody and PI. In brief, cells were harvested by trypsinization and fixed in 70% ethanol for 16-24 h. After washing in PBS, sequential incubation with anti-phospho-histone H3-Alexa 488 antibody (Cell Signaling Technology) and PI RNase staining buffer (BD Biosciences) was performed followed by flow cytometry. Classification of G1-, S-, G2-, and M-phase cells as well as apoptotic, subG1 cells is exemplarily shown in Suppl. Figure 1G.

### Quantitative realtime RT-PCR

RNA isolation and qRT-PCR were performed as described [76, 77]. Briefly, total RNA was extracted with the NucleoSpin RNA II Kit (Macherey & Nagel, Düren, Germany). 1 μg of RNA was reversely transcribed with 200 units RevertAid reverse transcriptase in the presence of 50 μM random hexamers, 5 μM Oligo(dT)_18_, 400 μM dNTPs, and 1.6 units/μl Ribolock RNase inhibitor (all from Thermo Scientific). The resulting cDNA (20 ng per reaction) was applied to qRT-PCR runs (20 μl final volume) with 300 nM primers (synthesized by Sigma-Aldrich) in 1x Maxima SYBR Green qPCR Mastermix (Thermo Scientific) and a standard cycling protocol (10 min 95°C, 45x (15 s 95°C, 30 s 60°C)) on an LC480 qPCR cycler (Roche). The following primer pairs were used: HSP70 Forward 5′-GAA GGA CGA GTT TGA GCA CAA GA-3′, HSP70 Reverse 5′-TGA TGA TGG GGT TAC ACA CCT G-3′, HSP90 Forward 5′-TTC AAA TTC ATC AGA TGC ATT GG-3′, HSP90 Reverse 5′-AAT ATG CAG CTC TTT CCC AGA GTC-3′, 18S rRNA Forward 5′-CGG CTA CCA CAT CCA AGG AA-3′, 18S rRNA Reverse 5′-GCT GGA ATT ACC GCG GCT-3′. Relative quantification was performed with the standard curve method, and the results were normalized on 18S rRNA. Untreated control cells served as calibrator.

### Syngeneic, heterotopic mouse model

In order to investigate the anti-tumor activity of NW457 as radiosensitizer and its tolerability *in vivo*, a syngeneic, heterotopic colorectal cancer model was used, where CT26 cells were transplanted on the right flanks of Balb/c mice. The experiments were approved by the *Regierung von Mittelfranken*. Throughout the studies, mice were provided a special diet and water *ad libitum* and were kept in well-ventilated cages under standard conditions of humidity (55 ± 5%), temperature (22 ± 2°C), and light (12/12 h light-dark cycles). Prior to injection, CT26 cells were trypsinized and washed in Ringer's solution. 1.28x 10^6^ CT26 cells were injected into the right flank of 8-week old female Balb/c mice (Janvier Labs, St. Berthevin, France) anaesthetized with isoflurane (Abbott Laboratories). Tumors were allowed to grow for 9 days consistent with an average tumor volume of approximately 200 mm^3^. Then, the tumor bearing mice were randomized into four experimental groups (vehicle only, NW457 only, 2x 5 Gy + vehicle, and 2x 5 Gy + NW457,). For *in vivo* studies NW457 was dissolved in DMSO as 100 mg/ml stock solution and stored at −20°C. Prior to injection, the stock solution was diluted with 0.5 vol of Tween-20 followed by 8.5 vol of 0.9% NaCl (37°C) to reach a final concentration of 10 mg/ml (10/5/85 DMSO/Tween-20/saline). The vehicle control (DMSO) was prepared accordingly. A fresh preparation was used for each dosing. 100 mg/kg NW457 or vehicle control, respectively, were injected intraperitoneally (i.p.) in a final volume of 500 μl on days 9, 12, 18, and 24 after tumor inoculation. Radiotherapy was performed on the basis of a CT-based treatment plan in order to ensure precise and selective irradiation of the tumor tissue and protection of the surrounding normal tissue as described previously [78]. Isoflurane-anaesthetized mice were positioned in a plexiglas box and irradiation was carried out on days 10 and 13 after tumor inoculation using a 6 MeV linear accelerator (ONCOR, Siemens, Erlangen, Germany) with a total dose of 5 Gy. Tumor dimensions were measured every two to three days with electronic digital calipers, and volumes were calculated using the formula (width x width x length)/2 [79]. When the tumor volume had reached 1,700 mm^3^, mice were sacrificed according to the guidelines of the Federation of European Laboratory Animal Science Associations (FELASA) and the Society of Laboratory Animals (GV-SOLAS). For the assessment of hepatotoxicity, serum levels of the liver enzymes ALT and AST were determined with the corresponding test kits (Biovision) according to the manufacturer's instructions. Additionally, routine histopathological examinations were performed on hematoxylin-eosin (HE)- and Giemsa-stained sections of formalin (10%)-fixed, paraffin-embedded liver tissue samples from 3-4 randomly selected mice per experimental group in a blinded fashion, i.e. without knowledge of the assignment of analyzed sections to the experimental/treatment group of mice.

### Statistics

If not stated otherwise, results are shown as means ± s.d. over 3-4 independent experiments (for *in vitro* experiments) and means ± s.e.m. over treatment groups (for *in vivo* experiments). *p*-values were calculated by unpaired Student's *t*-test analyses, two-way ANOVA, or log-rank test as indicated, and the threshold for statistical significance was set as *p* < 0.05.

Synergism between NW457 and irradiation was calculated by isobologram and combination index (CI) analyses. Isobolograms were constructed according to [33], where graphs of equally effective dose pairs (isoboles) for a single effect level are calculated. With a given effect level of the combination, doses of the single treatments that give an equal effect are computed and are plotted as axial points in a Cartesian plot. For non-linear dose-relationships a surface of additivity is constructed and the different types of interaction can be determined. Datapoints below the surface of additivity indicate a synergistic mode of action. Additionally, combination indices were calculated according to the median drug effect analysis method [34, 80]. This method calculates for each treatment pair a specific CI, which determines the degree of interaction between the analyzed therapy components, indicating either additivity (CI = 1), synergism (CI < 1), or antagonism (CI > 1).

## SUPPLEMENTARY MATERIAL



## Abbreviations

17-AAG: 17-(Allylamino)-17-demethoxy-geldanamycin
17-DMAG: 17-Dimethyl-amino-ethyl-amino-17-demethoxy-geldanamycin
Ac-DEVD-AMC: acetyl-aspartyl-glutamyl-valyl-aspartyl-amino-methyl-coumarine
ALT: alanine aminotransferase
AST: aspartate aminotransferase
ATM: ataxia telangiectasia mutated protein
ATR: ataxia telangiectasia and RAD3-related protein
Bax: Bcl-2-associated X protein
Bak: Bcl-2 homologous antagonist/killer
Bcl-xL: B-cell lymphoma-extra large protein
BRAF: V-Raf murine sarcoma viral oncogene homologue B1
BRCA1: breast cancer 1
BSA: bovine serum albumin
C12-FDG: 5-dodecanoylaminofluorescein-di-β-galactopyranoside
casp: caspase
CHK1: checkpoint kinase 1
CHK2: checkpoint kinase 2
CHX: cycloheximide
CI: combination index
DNA-PK: DNA-dependent protein kinase
DMSO: dimethyl sulfoxide
EGF: epidermal growth factor
EGFR: epidermal growth factor receptor
EPH: ephrin
FCS: fetal calf serum
FSC: forward scatter
GA: geldanamycin
γH2AX: phosphorylated histone H2AX
HE: hematoxylin-eosin
HSP: heat shock protein
i.p.: intraperitoneal
KRAS: kirsten rat sarcoma viral oncogene homologue
LC3A: Microtubule-associated proteins 1A/1B light chain 3A
MLKL: mixed lineage kinase domain-like protein
Noxa: phorbol-12-myristate-13-acetate-induced protein 1
p14ARF: tumor suppressor ARF
p21CIP1/WAF1: cyclin-dependent kinase inhibitor 1
p53: cellular tumor antigen p53
PARP: poly(ADP-ribose)polymerase
PI: propidium iodide
Puma: p53-upregulated modulator of apoptosis
qRT-PCR: quantitative realtime RT-PCR
RAD51: DNA repair protein RAD51 homologue 1
RIP kinase: receptor-interacting protein kinase
SSC: sideward scatter
STR: short tandem repeat
TNF: tumor necrosis factor
vol: volume
zVAD-fmk: benzyloxycarbonylvalyl-alanyl-aspartyl fluoromethyl ketone
