# Supplementary material for: A novel HSP90 inhibitor with reduced hepatotoxicity synergizes with radiotherapy to induce apoptosis, abrogate clonogenic survival, and improve tumor control in models of colorectal cancer
Source: Oncotarget. 2016 Jun 1;7(28):43199–219. doi: 10.18632/oncotarget.9774 (PMC5190018; doi:10.18632/oncotarget.9774)
Supplement: Supplementary file 1 [file oncotarget-07-43199-s001.pdf]

A novel HSP90 inhibitor with reduced hepatotoxicity synergizes with radiotherapy to induce apoptosis, abrogate clonogenic survival, and improve tumor control in models of colorectal cancer

Supplementary Material

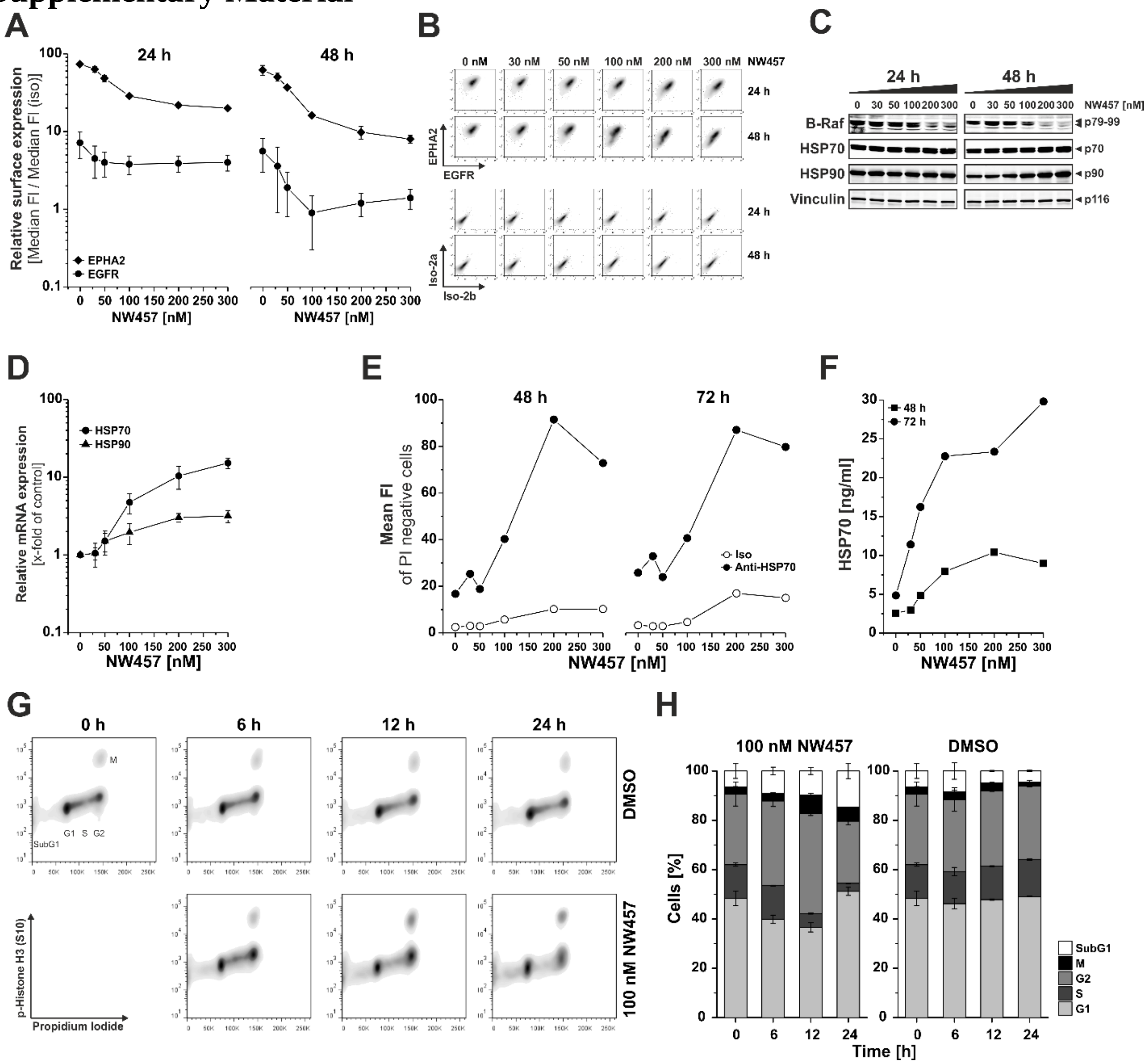

Supplemental Figure 1: HSP90 inhibition by NW457 leads to client protein degradation, upregulation of HSP90 and HSP70 expression, HSP70 surface exposure and release, but not to relevant shifts in cell cycle distribution.

HCT116 cells were treated with 0-300 nM NW457 for the indicated times, and HSP90 inhibition was assessed in terms of client protein degradation, upregulation of HSP90 and HSP70 expression, HSP70 surface exposure, and release. Cell cycle distribution was measured by anti-phospho-histone H3/PI FACS staining.

(A) Surface expression of the HSP90 client proteins ephrin A2 (EPHA2) and EGF receptor (EGFR) was measured by flow cytometry and is shown as median fluorescence intensities

normalized on the median fluorescence of the respective isotype controls. Means ± s.d. of three independent experiments are presented.

(B) Representative dot plots of (A) and appropriate isotype controls.

(C) Protein levels of BRAF, HSP70, and HSP90 were determined by Westernblot analysis of whole cell lysates (70 µg protein per lane). Vinculin (10 µg protein per lane) served as loading control.

(D) NW457-dependent induction of HSP70 and HSP90 mRNA expression was measured by quantitative real-time PCR 12 h after treatment. Relative expression levels normalized on 18S rRNA and calibrated on the vehicle control are shown as means ± s.d. of three independent experiments.

(E) Exposure of HSP70 on the cell surface was determined by anti-HSP70 FACS surface staining. Mean fluorescence intensity values of PI-negative cells are shown.

(F) NW457-dependent release of HSP70 was quantified by ELISA of HCT116 cell supernatants. Means of duplicates are depicted.

(G) Cell cycle distribution was assessed by anti-phospho-histone H3/PI FACS staining. Representative density plots depict how cell cycle phases were assigned.

(H) Quantification of the cell cycle data shown in (G). Means ± s.d. of triplicates are shown.

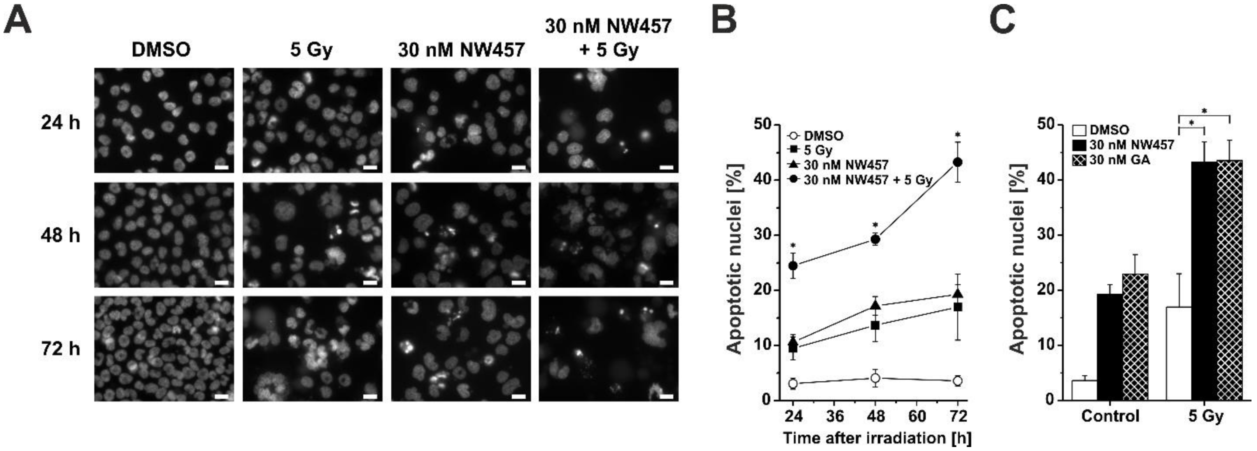

Supplemental Figure 2: NW457 induces chromatin condensation and nuclear fragmentation and sensitizes colorectal cancer cells to ionizing radiation.

HCT116 cells were treated with 30 nM NW457 or DMSO as vehicle control for 24 h, irradiated at 5 Gy, and apoptotic nuclei with signs of chromatin condensation and/or nuclear fragmentation were measured by Hoechst 33342 staining after 24-72 h.

(A) Nuclear morphology. Scale bars indicate 20 µm.

(B) Time course of apoptosis induction as quantified by Hoechst 33342 staining. Values represent means ± s.d. of three independent experiments. *$p < 0.01$ for combined treatment vs. irradiation or NW457 alone (unpaired Student's *t*-test).

(C) Apoptosis induction by NW457 in comparison to geldanamycin (GA). The percentage of cells with apoptotic nuclear phenotype in response to 30 nM NW457 or GA treatment alone or in combination with irradiation at 5 Gy was quantified 72 h after irradiation. Values represent means ± s.d. of three independent experiments. *$p < 0.01$ (unpaired Student's *t*-test).

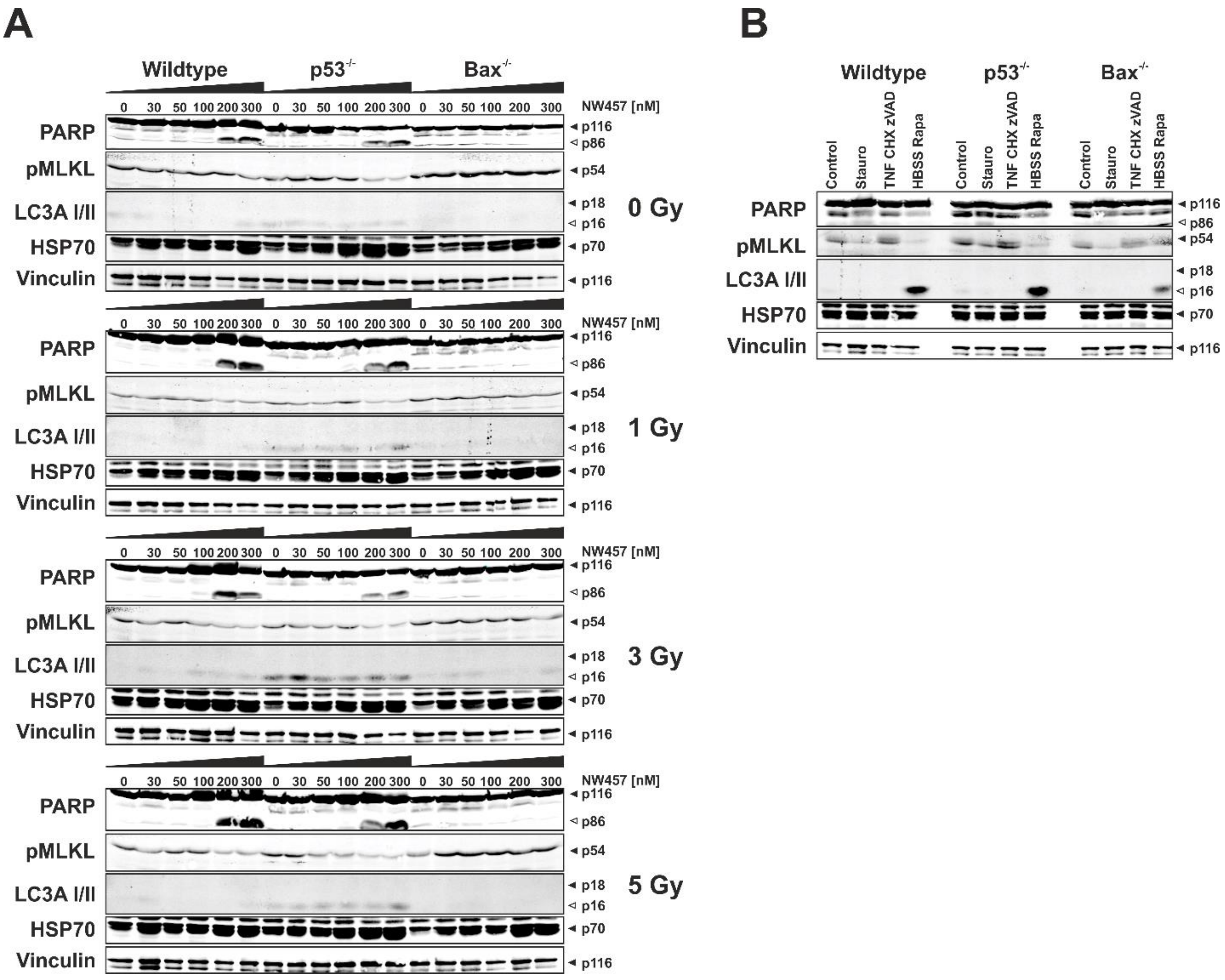

Supplemental Figure 3: Markers of necroptosis and autophagy cannot be detected in the context of NW457-mediated radiosensitization.

HCT116 wildtype cells and p53-deficient or Bax-deficient subclones were treated with 0-300 nM NW457 for 24 h, irradiated at 0-5 Gy, and markers of apoptosis (PARP cleavage), necroptosis (MLKL phosphorylation), and autophagy (LC3A conversion) were examined by Westernblot analyses 24 h after irradiation.

(A) Dose response relationship of changes in the respective marker proteins upon treatment with 0-300 nM NW457 and 0-5 Gy measured 24 h after irradiation.

(B) Positive controls for LC3A conversion (1 µM rapamycin in Hanks' buffered saline solution for 24 h), MLKL phosphorylation (50 ng/ml TNF, 100 µM cycloheximide, 50 µM zVAD-fmk for 24 h), and dephosphorylation of phospho-MLKL (5 µM staurosporine for 12 h).

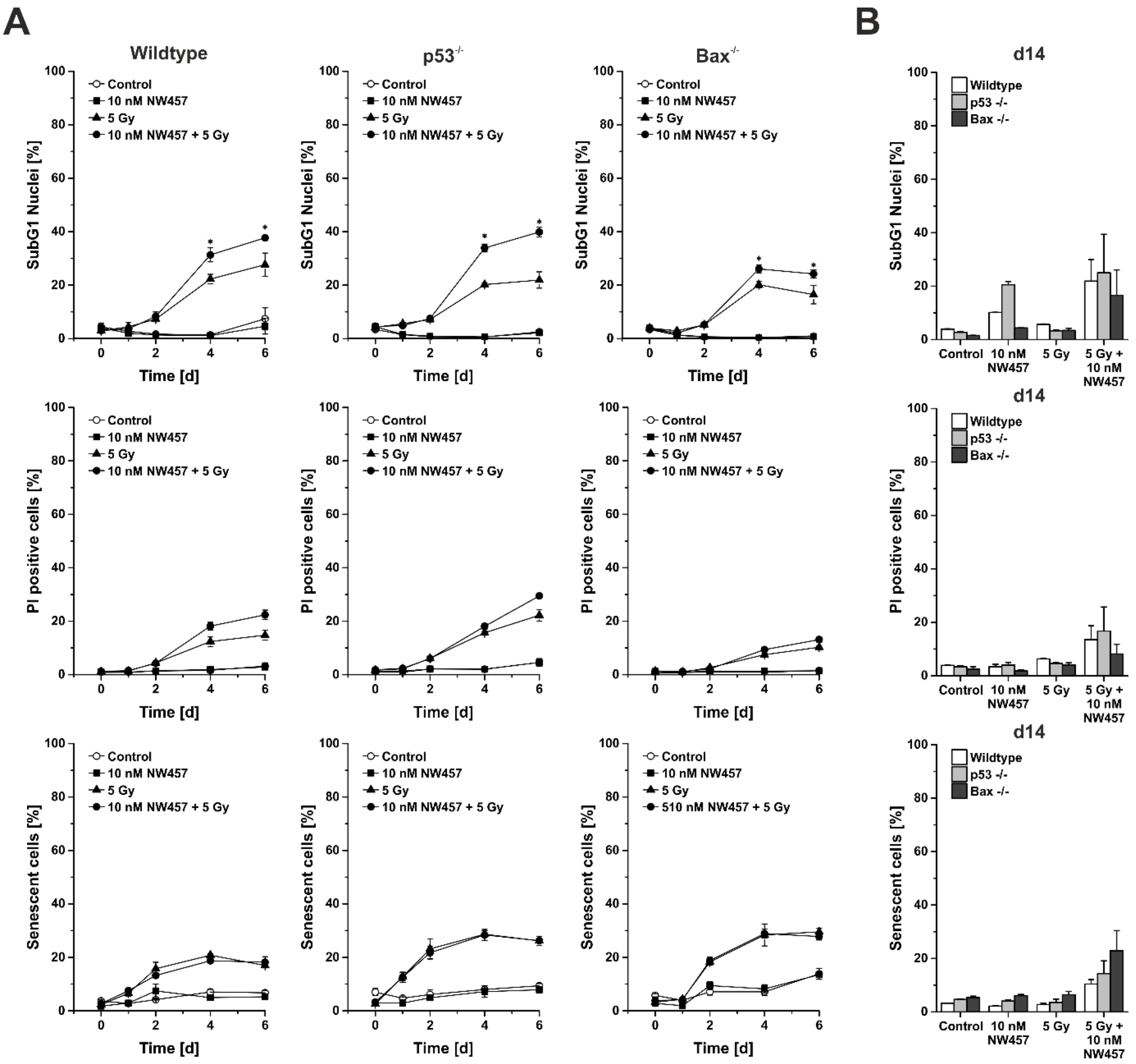

Supplemental Figure 4: Induction of apoptosis, necrosis, and senescence upon treatment with 10 nM NW457 in combination with irradiation is virtually independent of the p53 and Bax status.

HCT116 wildtype cells and p53-deficient or Bax-deficient subclones were treated with 10 nM NW457, irradiated at 5 Gy, and subjected to FACS analyses of subG1 nuclei (apoptosis), PI exclusion (necrosis), and beta-galactosidase activity (senescence).

(A) *Upper panel:* Analyses of subG1 nuclei on days 0-6 after irradiation. Means ± s.d. of triplicates are shown. **p* < 0.05 for combined treatment vs. irradiation only (unpaired Student's *t*-test). *Middle panel:* Measurement of necrosis on days 0-6 after irradiation (Means ± s.d. of triplicates). *Lower panel:* Assessment of senescence on days 0-6 after irradiation (Means ± s.d. of triplicates).

(B) Flow cytometric examination of apoptotic, necrotic, and senescent cells under the conditions of clonogenic survival assays with limiting cell numbers. Cells were pretreated with 10 nM NW457, irradiated at 5 Gy, and FACS analyses were performed on day 14 after irradiation. Means ± s.d. of triplicates are displayed.

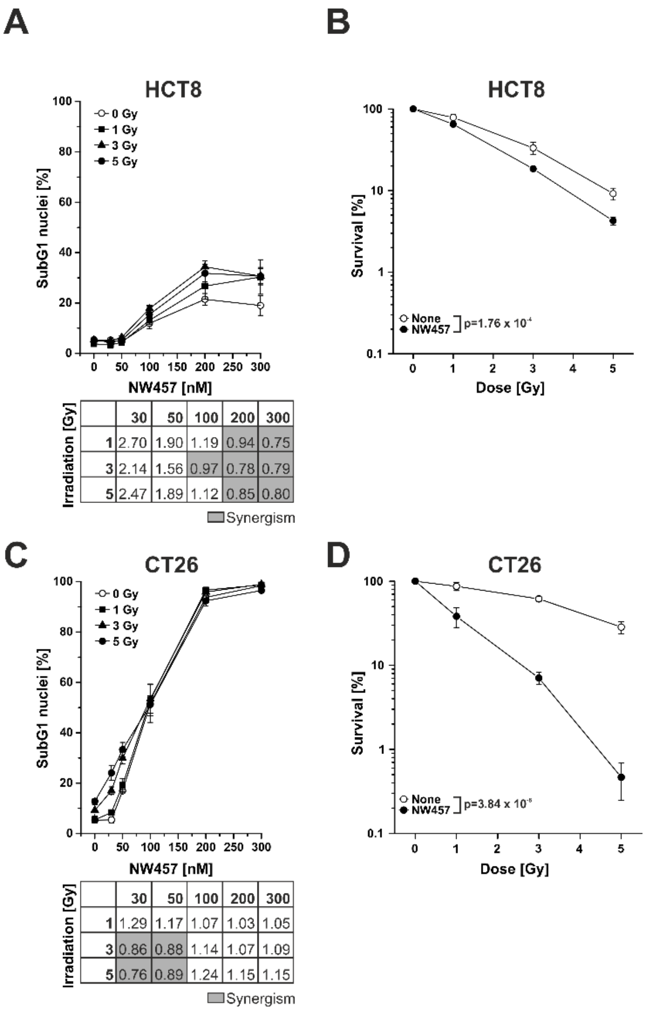

Supplemental Figure 5: NW457 synergizes with ionizing irradiation to induce apoptosis in HCT8 and CT26 cells and strongly enhances irradiation-induced clonogenic cell death.

HCT8 and CT26 cells were treated with 0-300 nM NW457 or DMSO as vehicle control for 24 h and irradiated at 0-5 Gy. Induction of apoptosis was determined by FACS analysis of subG1 nuclei 48 h after irradiation, and clonogenic survival was measured after 14 days.

(A, C) FACS analysis of subG1 nuclei 48 h after irradiation +/- NW457 treatment. CI matrices with synergistic combinations (CI < 1) highlighted in grey.

(B, D) Clonogenic survival. Cells were subjected to colony formation assays upon irradiation at 0-5 Gy +/- pretreatment with 10 nM NW457 for 24 h.
